# Transcription factor TaNF-YB2 interacts with partners TaNF-YA7/YC7 and transcriptionally activates distinct stress-defensive genes to modulate drought tolerance in *T. Aestivum*

**DOI:** 10.1186/s12870-024-05420-x

**Published:** 2024-07-25

**Authors:** Ying-Jia Zhao, Chun-Ying Ma, Meng-Jing Zheng, Yan-Rong Yao, Li-Hua Lv, Li-Hua Zhang, Xiao-Xin Fu, Jing-Ting Zhang, Kai Xiao

**Affiliations:** 1 Institute of Cereal and Oil Crops, Hebei Academy of Agriculture and Forestry Sciences/Hebei Key Laboratory of Crop Cultivation Physiology and Green Production, Shijiazhuang, 050035 P.R. China; 2 State Key Laboratory of North China Crop Improvement and Regulation, Hebei Agricultural University, Baoding, 071001 P.R. China; 3 College of Agronomy, Hebei Agricultural University, Baoding, 071001 P.R. China

**Keywords:** Wheat (*Triticum aestivum* L.), Nuclear factor-YB gene, Protein interaction; transcriptional activation, Transgene analysis, Stress-associated physiological parameter

## Abstract

**Background:**

Drought stress limits significantly the crop productivity. However, plants have evolved various strategies to cope with the drought conditions by adopting complex molecular, biochemical, and physiological mechanisms. Members of the nuclear factor Y (NF-Y) transcription factor (TF) family constitute one of the largest TF classes and are involved in plant responses to abiotic stresses.

**Results:**

*TaNF-YB2*, a NY-YB subfamily gene in *T. aestivum*, was characterized in this study focusing on its role in mediating plant adaptation to drought stress. Yeast two-hybrid (Y-2 H), biomolecular fluoresence complementation (BiFC), and Co-immunoprecipitation (Co-IP) assays indicated that TaNF-YB2 interacts with the NF-YA member TaNF-YA7 and NF-YC family member TaNF-YC7, which constitutes a heterotrimer TaNF-YB2/TaNF-YA7/TaNF-YC7. The *TaNF-YB2* transcripts are induced in roots and aerial tissues upon drought signaling; GUS histochemical staining analysis demonstrated the roles of *cis*-regulatory elements ABRE and MYB situated in *TaNF-YB2* promoter to contribute to target gene response to drought. Transgene analysis on *TaNF-YB2* confirmed its functions in regulating drought adaptation via modulating stomata movement, osmolyte biosynthesis, and reactive oxygen species (ROS) homeostasis. TaNF-YB2 possessed the abilities in transcriptionally activating *TaP5CS2*, the P5CS family gene involving proline biosynthesis and *TaSOD1*, *TaCAT5*, and *TaPOD5*, the genes encoding antioxidant enzymes. Positive correlations were found between yield and the *TaNF-YB2* transcripts in a core panel constituting 45 wheat cultivars under drought condition, in which two types of major haplotypes including TaNF-YB2-Hap1 and -Hap2 were included, with the former conferring more *TaNF-YB2* transcripts and stronger plant drought tolerance.

**Conclusions:**

*TaNF-YB2* is transcriptional response to drought stress. It is an essential regulator in mediating plant drought adaptation by modulating the physiological processes associated with stomatal movement, osmolyte biosynthesis, and reactive oxygen species (ROS) homeostasis, depending on its role in transcriptionally regulating stress response genes. Our research deepens the understanding of plant drought stress underlying NF-Y TF family and provides gene resource in efforts for molecular breeding the drought-tolerant cultivars in *T. aestivum*.

**Supplementary Information:**

The online version contains supplementary material available at 10.1186/s12870-024-05420-x.

## Introduction

Drought stress during plant growth and development is one of the adverse environmental factors, leading to a drastic reduction on plant productivity of major crops [1, 2]. On the other hand, plants have evolved a suite of strategies, such as escape, avoidance, and tolerance to drought stress, to enhance their capacities for survival and biomass production under conditions challenged by drought [3]. These processes in plant drought responses are accomplished based on the complicated mechanisms that reprogram a wide range of pathways integrated at the molecular, biochemical and physiological levels [4].

In past two decades, a subset of physiological processes associated with plant drought response has been defined. At early stage of water deficit, plants usually enhance the abilities for water taken up from growth media through enlarged root system, reduction of water loss from transpiration by promoting stomata closing, and acclimation of biochemical metabolisms to adjust available carbon resources in plants [5–7]. Under intensified drought conditions, some kinds of osmolytes, such as proline, soluble sugar, spermine, and betaine, are induced for biosynthesis to maintain suitably cellular turgor pressure [8, 9]. In addition, reactive oxygen species (ROS) homeostasis-associated parameters, including the activities of antioxidant enzymes (AE) such as superoxide dismutase (SOD), catalase (CAT), and peroxidase (POD), are altered in plants challenged by drought stress [7, 10]. These plant responses are associated closely with the drought signal transduction systems, mainly those of abscisic acid (ABA)-dependent and -independent pathways through integrating transcription of a quantity of genes, such as those related to stress signal transducing, gene transcriptional regulation, and transcription behaviors at downstream processes of the signaling systems, which function synergistically and contribute to the plant tolerance to drought stressor.

NUCLEAR FACTOR Y (NF-Y) proteins constitute a large group of transcription factor (TF) families in eukaryotes and are specified by a motif referred to as CCAAT-binding box (CBF) [11, 12]. A NF-Y TF is present in plants as a heterotrimer that composes three kinds of subunits, including NF-YA (also named CBF-B or HAP2), NF-YB (CBF-A or HAP3), and NF-YC (CBF-C or HAP5). Investigations have indicated that each of the NF-Y family subunit contains conserved domains that are involved in binding DNA and in interacting with distinct proteins [12]. Although the subunits of NF-Y proteins are encoded by single gene in mammals and yeast, each subunit in plant species is encoded by a family with a subset of genes. For example, in total of 10, 13, and 13 genes encoding subunits NF-YA, NF-YB and NF-YC, respectively, are found in the genome of model plant Arabidopsis [13]; 11, 11, and 12 genes encoding subunits NF-YA, NF-YB and NF-YC are identified in genome of rice [13]; and 18, 34, and 28 genes encoding subunits of NF-YA, NF-YB and NF-YC are documented in genome of wheat (*T. aestivum*) [14]. The findings on the multiplicity nature of the NF-Y family genes suggest that they are functional in constituting a large number of heterotrimers through putatively diverse combinations among them after gene transcription and translation [15]. Among these NF-Y proteins, subunit NF-YA is specified by its role in binding with CCAAT box [11, 12]; subunits NF-YB and NF-YC display conserved histone fold motifs that promote tight dimer establishment between them, based on the results from the crystal structure analyses [12]. The dimer established by NF-YB and NF-YC further interact with subunit NF-YA through a histone H2A-H2B dimer that involves binding to DNA element [12].

Functions of NF-Y TF in mediating plant growth and development, such as root growth, chloroplast biogenesis, fatty acid biosynthesis, flowering time and embryo genesis, have been well documented [16, 17]. Moreover, an array of investigations on NF-Y family members has also confirmed their roles in regulating plant abiotic stress responses. For example, the *AtNF-YA5* transcripts in Arabidopsis are strongly induced upon drought with an ABA-dependent manner; its overexpression elevates plant drought tolerance by inducing transcription of a number of drought stress-responsive genes [18]. Under nitrogen (N) starvation condition, the transcript levels of *AtNF-YA2*, *3*, *5*, *7*, and *10* in Arabidopsis plants are increased by 5- to 50-fold. Additionally, the transcripts of *AtNF-YA3* and *5* increased by two-fold after salt treatment. Overexpression of *AtNF-YA2*, *7* and *10* enhances plant tolerance to several types of abiotic stresses, including flooding, N starvation, freezing, and high temperature, through their functions to modulate the transcription efficiencies of stress responsive-associated genes [19]. A set of members in NF-YB subfamily, including Arabidopsis *AtNF-YB1*, maize *ZmNF-YB2*, and poplar *PdNF-YB7*, all confer plants an improved osmotic stress acclimation by enhancing plant water-use efficiency [20]. In contrast to above NF-Y genes that positively affect plant stress responses, *AtNF-YA1* in Arabidopsis exerts negative roles in regulating seed germination and post-germination growth behaviors under salt stress [21]. Similarly, *OsNF-YA2* in rice acts as one negative regulator in plant abiotic stress tolerance. These findings together suggest the extensive effects of NF-Y TFs in plant responses to adverse abiotic stressors.

Thus far, although a large set of investigations have been extensively conducted on members of the NF-Y family in plants, especially in the model plant species [22], characterizations on the genes of NF-Y family (i.e., those encoding subunits NF-YA, NF-YB, and NF-YC) are still limited in *T. aestivum*, a major crop cultivated extensively worldwide. In this study, we characterized *TaNF-YB2*, a member of the NF-YB subfamily situated on chromosome 2B of wheat, in mediating plant response to drought stress. Our results indicated that *TaNF-YB2* responds sensitively to drought signaling at transcriptional level and contributes largely to plant drought adaptation, through its roles in transcriptionally regulating a suite of stress-defensive genes, which are involved in the modulation of stomata movement, leaf water retention capacity, osmolyte accumulation, and ROS homeostasis.

## Materials and methods

### Characterization analysis on *TaNF-YB2*

*TaNF-YB2* (accession No. TraesCS2B02G199800.1) is situated on chromosome 2B and shares high similarity to *NF-YB2* TF members in plant species. It displayed significantly upregulated transcripts in root tissues of Shimai 26 (drought-tolerant cultivar) upon drought signaling in our previous RNA-seq analysis (data to be published), which prompted us to characterize it in more detail. The molecular weight (mW) and isoelectric point (pI) of TaNF-YB2 protein were predicted based on the software DNAStar (https://www.dnastar.com). To generate a phylogenetic tree of *TaNF-YB2* with its counterparts in diverse plant species, we firstly identified its homologous genes based on BLASTn search analysis against the GenBank database of NCBI, using its cDNA sequence as a query. The phylogenetic relations among *TaNF-YB2* and its homologous genes across various plant species were then established based on MEGA7 software. Multiple alignment analysis was performed for TaNF-YB2 and the plant counterparts using the MegAlign algorithm supplemented in software DNAStar (https://www.dnastar.com). The three-dimensional structure of TaNF-YB2 protein was modeled by adopting an online tool referred to as SWISS-MODEL algorithm (https://swissmodel.expasy.org/interactive), using the related prediction program as suggested.

An experiment was conducted to define the subcellular localization of TaNF-YB2 after endoplasmic reticulum (ER) assortment. With this purpose, an expression cassette harboring fusion *TaNF-YB2* and a reporter gene *green fluorescent protein* (*GFP*) (*TaNF-YB2-GFP*) was constructed and subjected to genetic transformation of model plant *N. benthamiana* and wheat protoplasts as described by Guo et al. (2013) [6], using gene specific primers for amplification of the open reading frame (ORF) (Table S1). GFP signals from transgenic cells initiated by fusion TaNF-YB2-GFP were detected under fluorescent microscope (Olympus FV10-ASW, Japan). During which, a specific nuclei staining using DAPI (Solarbio, Beijing, China) was acted as a positive control for nucleus localization performed by following the manufacturer’s instructions.

### Expression pattern analysis of *TaNF-YB2* upon drought stress

Expression patterns of *TaNF-YB2* together its homeologs situated on other two chromosomes, namely, 2 A (*TaNF-YB2-2A*, TraesCS2A02G173500) and 2D (*TaNF-YB2-2D*, TraesCS2D02G180700), were analyzed under drought conditions, using roots and leaves of wheat (cv. Shimai 26) seedlings as materials. Firstly, the seeds were germinated in a growth chamber and the young seedlings were cultured in a standard Murashige and Skoog (MS) solution as described previously [23]. The wheat seedlings were then subjected to drought treatment at the third leaf stage by culturing in a modified MS solution supplemented with polyethylene glycol 6000 (10% PEG-6000, w/v), a kind of osmotic chemicals generally used for plant simulated drought treatment. The tissues mentioned were sampled at 0 h (before treatment) and 1, 3, 9, and 27 h following the drought treatment. Transcripts of *TaNF-YB2* in tissues were evaluated based on qRT-PCR. Moreover, an aliquot of a 27 h drought-challenged seedlings were re-grown in the standard MS solution and tissues were sampled at 1, 3, 9, and 27 h following the recovery treatment, by which to understand the gene response to normal recovery condition. Total RNA in collected samples was extracted using TRIzol reagents (Invitrogen) following the manufacturer’s instruction. The first-strand cDNA was synthesized from total RNA using PrimerScript RT Master Mix (TaKaRa, China) and used as the template in qRT-PCR analysis. The transcripts of *TaNF-YB2* and its homeologs (i.e., *TaNF-YB2-2A* and *TaNF-YB2-2C*) were detected using a two-step reaction with reagent (PowerUP SYBRGreen Master Mix) and 7500 Real-Time PCR System (Applied Biosystems, USA). PCR constituents comprised a 25 µl volume containing following components: 12.5 µl KOD FX PCR buffer (TaKaRa, China), 5 µl dNTPs, 0.75 µl each of 10 pmol forward and reverse primers, 2.5 µl template cDNA, 3 µl ddH_2_O, and 0.5 µl KOD FX (TaKaRa, China), using a following thermal cycling program: 94 °C for 2 min, then 25 cycles of 94 °C for 30 s, 55 °C for 30 s, and 68 °C for 45 s, followed by a final extension at 68 °C for 8 min. The constitutive genes *Tatubulin* and *TaGAPDH* in *T. aestivum* were used as internal standards to normalize the transcripts of target genes with gene specific primers (Table S1).

### GUS staining analysis to define roles of *cis*-elements in *TaNF-YB2* promoter

An online search analysis identifying the putative *cis*-acting regulatory elements in *TaNF-YB2* promoter was performed using PLACE (http://bioinformatics.psb.ugent.be/webtools/plantcare/html/). To characterize the roles of a set of *cis*-acting elements putatively involving transcriptional regulation of genes upon drought response, several truncated fragments across a 2-kb promoter, including lengths of 263, 591, 1112, 1469, and 1771 bp upstream the translation start site ATG, which contained elements associated with gene transcription regulation and osmotic stress response (i.e., ABRE, and recognition sites MYB and MYC), were PCR amplified using specific primers (Table S1). They were then separately inserted in front of the reporter gene (i.e., *green fluorescent protein*, *GFP*) in binary vector pCAMBIA3301, and further subjected to genetic transformation onto *A. tumefaciens* strain EHA105 using a heat-shock approach as described by [24]. The positive transformants harboring empty vector (control) and various cassettes were then used to transform young embryos of wheat (Shimai 22) based on an *Agrobacterium*-mediated transformation method as previously described [23]. Over 5 independent transgenic lines for each construct were generated. The seedlings of the transgenic lines integrated various truncated *TaNF-YB2* promoter regions and control were cultured normally in an MS solution to the third leaf stage. They were then subjected to simulated drought treatment (supplemented with PEG-6000, w/v, 10%) for 6 h. GUS activities of the transgenic lines and control were assessed based on a histochemical staining approach by staining representative infiltrated leaves for 24 h at 37 °C in a GUS staining solution, which comprised following constituents: 0.1% Triton X-Gluc, pH 7.2, and 10 mM EDTA. The GUS staining features in samples were recorded using a digital camera after several wash processes using graded ethanol series. The activities of GUS in samples were assessed following the method as previously described [25].

### Detection of partners on NF-YA and NF-YC members in constituting heterotrimer with TaNF-YB2

Experiments on yeast two-hybrid (Y-2 H), bimolecular florescence complementation (BiFC) and Co-immunoprecipitation (Co-IP) assays were conducted to understand the protein–protein interactions between TaNF-YB2 and its counterparts in NF-YA and NF-YC subfamilies. Of which, in Y-2 H assays, the TaNF-YB2 protein was expressed in *S. cerevisiae* host strain AH109 and acted as a bait whereas the proteins of subfamily NF-YA (i.e., *TaNF-YA2*, *TaNF-YA3*, and *TaNF-YA7*) or those in subfamily NF-YC (i.e., *TaNF-YC1* to *TaNF-YC8*) expressed separately in the yeast host strain acted as preys. The full-length coding sequences of *TaNF-YB2* and those of NF-YA/NF-YC members integrated into the restriction sites of vectors pGBKT7 or pGADT7 were PCR amplified, using wheat cDNA as template and gene specific primers (Table S1). Positive transformants harboring both bait and prey that indicated protein-protein interactions between TaNF-YB2 and its NF-YA or NF-YC members were detected by culturing them on a selecting medium lacking distinct amino acids, namely, SD/-Leu/-Trp, with a culture duration of 3 d under 30 °C condition. The auto-activation of the NF-Y transcription factors was measured by assessing the expression β-galactosidase reporter genes in the yeast strain growing on selective media, together with a positive auto-activation marker.

BiFC assays were conducted to further validate the protein interactions between TaNF-YB2 and its counterparts. To this end, ORFs of the target genes were RT-PCR amplified using specific primers (Table S1) and fused with fragments of the reporter gene *YFP* (yellow fluorescent protein encoding gene) at the C- or N-terminus as previously described [26]. The resulting constructs were then transiently co-transformed onto *N. benthamiana* leaves using *Agrobacterium* (stain EHA105)-mediated infiltration method [27]. The YFP signals in transgenic leaves after 2 d of infiltration were detected under a fluorescent microscope (Olympus FV10-ASW, Japan) following the manusfactuer′s suggestions. In parallel to YFP signal detection, the nuclei in epidermal cells of *N. benthamiana* were stained using DAPI (Solarbio, Beijing, China) that acted as a positive control showing the protein nucleus localization as previously described [28].

Co-IP assays were also performed to validate the protein interaction among TaNF-YB2 and its NF-YA and NF-YC partners. With this purpose, the ORFs of *TaNF-YB2* and its partners, namely TaNF-YA7, and TaNF-YC7 together with cDNA sequences encoding distinct segments of above signaling members (TaNF-YB2, TaNF-YA7, and TaNF-YC7), were amplified based on RT-PCR using gene-specific primers (Table S1). They were then subjected to construction of expression cassettes, namely MET-TaNF-YB2/TaNF-YB2-GFP, MET-TaNF-YA7/TaNF-YA7-GFP, and MET-TaNF-YC7/TaNF-YC7-GFP, and used to transform *A. tumefaciens* stain EHA105. Positive transformants were then subjected to transient transformation of *N. benthamiana* leaves as previously described [29, 30]. Briefly, approximately 0.3 g of leaves were collected after 24 h infiltration and ground into powder using 1 mL of buffer A (50 mM HEPES-NaOH pH 7.5, 150 mM NaCl, 5% glycerol, 0.5% Triton X-100, 0.1% (v/v) 3-mercapto-1, 2-propanediol, 1 tablet of complete mini protease inhibitor cocktail (Shangon, Shanghai, China) per 10 mL). The resulting solutions were centrifuged twice at 1000 g, 4 °C for 10 min to obtain the protein components. They were further mixed with an equal volume of 2×sodium dodecyl sulfate-polyacrylamide gel electrophoresis (SDS-PAGE) sample buffer [(Laemmli sample buffer (BIO-RAD, California, USA) 950 µl/mL, 3-mercapto-1, 2-propanediol 50 µl/mL)], and denatured at 95 °C for 5 min then stored at 30 °C. The proteins were then incubated with GFP-trap agarose beads (Chromotek, Planegg, Germany) for protein binding at 4 °C. After 4–6 time washes of beads with buffer A, the proteins were eluted with 1×SDS-PAGE sample buffer (50% 2×SDS-PAGE sample buffer, 50% 1×PBS). Co-immunoprecipitation assays were conducted using a 12.5% SDS-PAGE gel (e-PAGEL) (ATTO, Tokyo, Japan).

### Generation of *TaNF-YB2* transgenic lines

Transgenic wheat lines for *TaNF-YB2* were generated to investigate its function in mediating drought response. Briefly, its ORF was amplified in both sense and anti-sense orientations using gene-specific primers (Table S1) through RT-PCR. The amplified products were then separately integrated into the *Nco*I/*BstE*II sites of binary vector pCAMBIA3301 under the control of the CaMV35S promoter. These expression cassettes were then used to genetically transform *A. tumefaciens* (strain EHA105). Positive transformants were selected and used to transform *T. aestivum* (cv. Shimai 22) using previously described method [23]. The transcripts of target gene in transgenic lines were evaluated through qRT-PCR using gene-specific primers (Table S1).

### Determination of stress-associated physiological indices for *TaNF-YB2* transgenic lines

Two typical lines with overexpression (integrated in sense orientation) and another two ones with knockdown expression (integrated in anti-sense orientation) for each gene at the T3 generation (Sen 2, Sen 3, Anti 1, and Anti 2) together with the wild type (WT), were cultured under normal condition and subjected to drought treatment under a field condition. The experiment was conducted at the Experimental Station of Hebei Agricultural University during 2021–2022 growth season. For normal growth condition, four irrigations (i.e., irrigated before seed-sowing, and jointing, flowering and mid-filling stages) were provided, with 60 mm water amount applied each controlled using a water amount analyzer. The drought treatment was established by reduced irrigations during the growth season, with two irrigations performed (before seed-sowing and jointing stage) with 60 mm of water supplied at each irrigation. The transgenic lines of *TaNF-YB2* (Sen 2, Sen 3, Anti 1, and Anti 2) together with WT were planted in two rows (2 m in length and 2 rows with row distance of 15 cm), with three replicates for normal growth and drought treatment. The seeds were sown on October 8, 2021 with seed amount 150 kg/ha. Basal complex fertilizer (N: P_2_O_5_: K_2_O/18: 12: 16) at rate of 600 kg/ha and topdressed nitrogen (source of urea, 120 kg/ha N) at jointing stage along with irrigation was applied to the transgenic lines and WT. Other managements such as chemical control for weeds and diseases were similar to those in local winter wheat production.

At seed filling stage, the growth phenotypes and plant biomass for transgenic lines and WT were analyzed under normal condition or drought treatment. Of which, the phenotypes were recorded using images taken by a digital camera. Plant biomass were obtained from ten representative plants after oven-drying following the conventional approach. In addition, the chlorophyll contents in upper leaves (top two leaves of plants) were assessed using a analyzer (SPAD-502). At maturity, the seeds were subjected to phenotype recordation and assessment of grain weight after seed air-drying. Plant biomass for transgenic lines and WT was obtained from twenty representative plants and yields in plots were measured based on air drying grain weights of 0.5 m^2^ plants.

Aside from measurements of plant growth and agronomic traits as well as chlorophyll contents, a set of osmotic stress-associated physiological indices were evaluated in the transgenic lines of *TaNF-YB2* and WT under normal condition and drought treatment. The assessed indices included stomata closing reate, leaf water losing rate (WLR), contents of osmolytes (proline and soluble sugar), and reactive oxygen species (ROS) homeostasis parameters (activities of SOD, CAT, and POD, contents of MDA, and amounts of superoxide anion and H_2_O_2_). Among these, the stomata closing rates were determined by recording stomata widths across a 1 h-regime drought treatment (0.25, 0.5, and 1 h) compared with that at time point 0 h (before treatment), following the approach as previously described [23]; leaf WLR values were calculated by losed fresh weights of detached leaves under a 3 h-regime water deficit treatment (0.5, 1, and 3 h) by putting leaves on a clean bench; the contents of osmolytes, including proline and soluble sugars acting as bio-markers for plant osmotic regulatory activity, were assessed as described previously [31, 32]; the activities of SOD, CAT, and POD, contents of MDA were determined using the methods described previously [33], and amounts of superoxide anion and H_2_O_2_ were evaluated using histochemical staining methods, namely nitro blue tetrazolium (NBT) staining for superoxide anion and 3.3*’*-diaminobenzidine (DAB) staining for H_2_O_2_ [23].

### Characterization of expression patterns and functions of P5CS and AE genes in mediating drought responses

The genes encoding P5CS that act as limiting step enzyme for proline biosynthesis and those encoding AE proteins impacting cellular ROS scavenging were subjected to expression analysis under drought treatment, by which to understand the molecular processes underlying *TaNF-YB2*-mediated osmolytes accumulation and ROS homeostasis. The P5CS genes and AE genes were identified in NCBI GenBank database. In total of 5 P5CS genes (i.e., *TaP5CS1* to *TaP5CS5*), 6 SOD genes (i.e., *TaSOD1* to *TaSOD6*), 6 CAT genes (i.e., *TaCAT1* to *TaCAT6*), and 9 POD genes (i.e., *TaPOD1* to *TaPOD9*) were subjected to analysis of expression levels based on qRT-PCR using gene specific primers (Table S1).

Yeast one-hybrid assays were performed to verify the interaction of TaNF-YB2 on its targets, namely *TaP5CS2*, *TaSOD1*, *TaCAT5*, and *TaPOD5* following the previous method [11]. With this purpose, the *TaNF-YB2* ORF was RT-PCR amplified using gene-specific primers (Table S1) and integrated into pGADT7 vector (Clontech) at *BamH*I and *Pst*1 sites to generate fusion pGADT7-*TaNF-YB2* (as prey). At the meantime, the promoter regions of its target genes were separately inserted into pHIS2 reporter vector (Clontech) at *Nco*I and *BstE*II site to generate fusions of pHIS2-TaP5CS2_Pro_, pHIS2-TaSOD1_pro_, pHIS2-TaCAT5_pro_, and pHIS2-TaPOD5 _pro_ (as baits). The combinations consisting of prey and each bait plasmid were co-transformed into yeast strain (EGY48). Positive transformants were identified after 3 d culture on solid selection growth media lacking SD-Trp/ His/-Leu.

Transient expression assays characterizing the transcriptional activation of *TaP5CS2*, *TaSOD1*, *TaCAT5* and *TaPOD5* underlying TaNF-YB2, TaNF-YA7 and TaNF-YC7 were performed in *N. benthamiana* leaves, given that these stress response genes displayed modified expression in *TaNF-YB2* transgenic lines after drought treatment. With this purpose, the ORFs of wheat NF-Y TF genes (i.e., *TaNF-YB2*, *TaNF-YA7*, and *TaNF-YC7*) were RT-PCR amplified using gene-specific primers (Table S1) and integrated in binary cassette to generate effectors including CaMV35S_pro_::*TaNF-YB2*, CaMV35S_pro_::*TaNF-YA7*, CaMV35S_pro_::*TaNF-YC7*. At the mentime, the promoter regions (2 kb in length) of *TaP5CS2*, *TaSOD1*, *TaCAT5*, and *TaPOD5* were PCR amplified using wheat (Shimai 26) genome DNA as a template and specific primers (Table S1), then integrated in frame with reporter gene *LUC*. The promoter-*LUC* fusions were inserted separately into the pGreenII 0800-LUC vector to generate four reporters, namely TaP5CS2_pro_::*LUC*, TaSOD1_pro_::*LUC*, TaCAT5_pro_::*LUC* and TaPOD5_pro_::*LUC*. Following binary cassette combinations consisting of effector(s) and reporter(s) were established, including effectors CaMV35S_pro_::*TaNF-YB2*, CaMV35S_pro_::*TaNF-YA7*, CaMV35S_pro_::*TaNF-YC7*, CaMV35S_pro_::*TaNF-YA7*/*TaNF-YB2*/*TaNF-YC7* and reporter TaP5CS2_pro_::*LUC*; effectors and reporter TaSOD1_pro_::*LUC*; effectors and reporter TaCAT5_pro_::*LUC*; and effectors and reporter TaPOD5_pro_::*LUC*. The effector/reporter combinations were separately co-transformed onto *A. tumefaciens* strain EHA105 as described previously using a transformant cell mixture of a ratio 1:1 (v: v). Positive transformants were further subjected to transient transformation of epidermal cells of *N. benthamiana* [31]. After 48 h of transformation and spraying 100 mM luciferin, the reporter fluorescein signals in transiently transformed cells were detected using a charge-coupled device imaging apparatus (NightOWL II LB983 in conjunction with Indigo software) according to the manufacturer′s instruction with six biological replicates.

Transgene analysis on the stress-associated genes, namely *TaP5CS2*, *TaSOD1*, *TaCAT5*, and *TaPOD5* was conducted to verify their roles in mediating plant drought response. With this purpose, the ORFs of above genes were amplified based on RT-PCR in anti-sense orientation using gene specific primers (Table S1). The transgenic lines with knockdown expression of them (Fig. S9) were generated to be similar to the lines with knockdown expression of *TaNF-YB2* mentioned above. Two typical transgenic lines for each gene, including AntiP5CS2-1 and AntiP5CS2-2 for *TaP5CS2*, AntiSOD1-2 and AntiSOD1-3 for *TaSOD1*, AntiCAT5-1 and AntiCAT5-2 for *TaCAT1*, and AntiPOD5-2 and AntiPOD5-3 for *TaPOD5*, were subjected to drought treatment established as aforementioned. The transgenic lines and WT were subjected to normal growth and drought treatment cultivated under field condition to be similar to the *TaNF-YB2* lines as aforementioned. At mid-seed filling stage, the proline contents and biomass in lines with *TaP5CS2* knockdown were assessed whereas the AE activities and MDA contents in lines with knockdown expression of the AE genes, namely, *TaSOD1*, *TaCAT5*, and *TaPOD5*, were evaluated similarly to those in the *TaNF-YB2* transgenic lines.

### Assessment of gene transcripts, agronomic traits, and haplotype properties in wheat cultivar panel

A wheat variety panel consisting of 45 wheat cultivars differed in drought response was used to evaluate the relationship between agronomic traits and transcripts/SNPs of *TaNF-YB2* under drought cultivation condition. The cultivar information, such as names, breeding institutes, and registration times, are listed in Table S2. To this end, a field experiment was conducted at the Experimental Station of Hebei Agricultural University, Baoding, during the 2022–2023 growth season, using a random block design for tested wheat cultivars with triplicate (5 m in length and 3 m in width). During the whole growth circle, two irrigations, namely those prior to seed-sowing and jointing stage that are considered as deficit irrigation management for local winter wheat (with average − 1.05 mPa of soil water potential at ploughing layer), were applied. At mid-filling stage, upper leaf samples (i.e., top two leaves of plants) from the tested cultivars under soil drought stress (-1.05 mPa of soil water potential at the ploughing layer) were collected and used to evaluate transcripts of *TaNF-YB2*, *TaP5CS2*, *TaSOD1*, *TaCAT5* and *TaPOD5* based on qRT-PCR using gene-specific primers (Table S1). The constitutive genes *Tatubulin* and *TaGAPDH* were used as internal standards to normalize the target transcripts. At maturity, the yields for the tested wheat cultivars were measured based on the air drying grain weights of 5 m^2^ plants.

The putative haplotypes of *TaNF-YB2* were characterized using this core wheat variety panel. With this aim, the promoter regions of *TaNF-YB2* were amplified from all of the tested wheat cultivars and were subjected to sequencing analysis. The haplotype types were defined based on the behaviors of the single nucleotide polymorphism (SNP) and the drought tolerance in term of yield.

### Statistical analysis

Means of gene expression levels detected by qRT-PCR, osmotic stress-associated physiological parameters, such as stomata closing rate, WLR in detached leaves, osmolyte contents, the ROS-associated parameters, chlorophyll contents, plant biomass, and yields in transgenic lines and WT as well as wheat cultivars under normal condition and drought treatment, were derived from the results of three replicates. Standard errors of the means and significant differences among the assayed traits shown in different treatments and transgenic lines with respect to WT, and regression analysis results between the yield and expression levels of *TaNF-YB2*, *TaP5CS2*, *TaSOD1*, *TaCAT5* and *TaPOD5* in a wheat variety panel under the drought field conditions, were assessed based on the analysis of variance (ANOVA) and Student′s *t*-test, with a critical value of *P* ≤ 0.05.

## Results

### Molecular characterization of *TaNF-YB2*

**Fig. 1:**
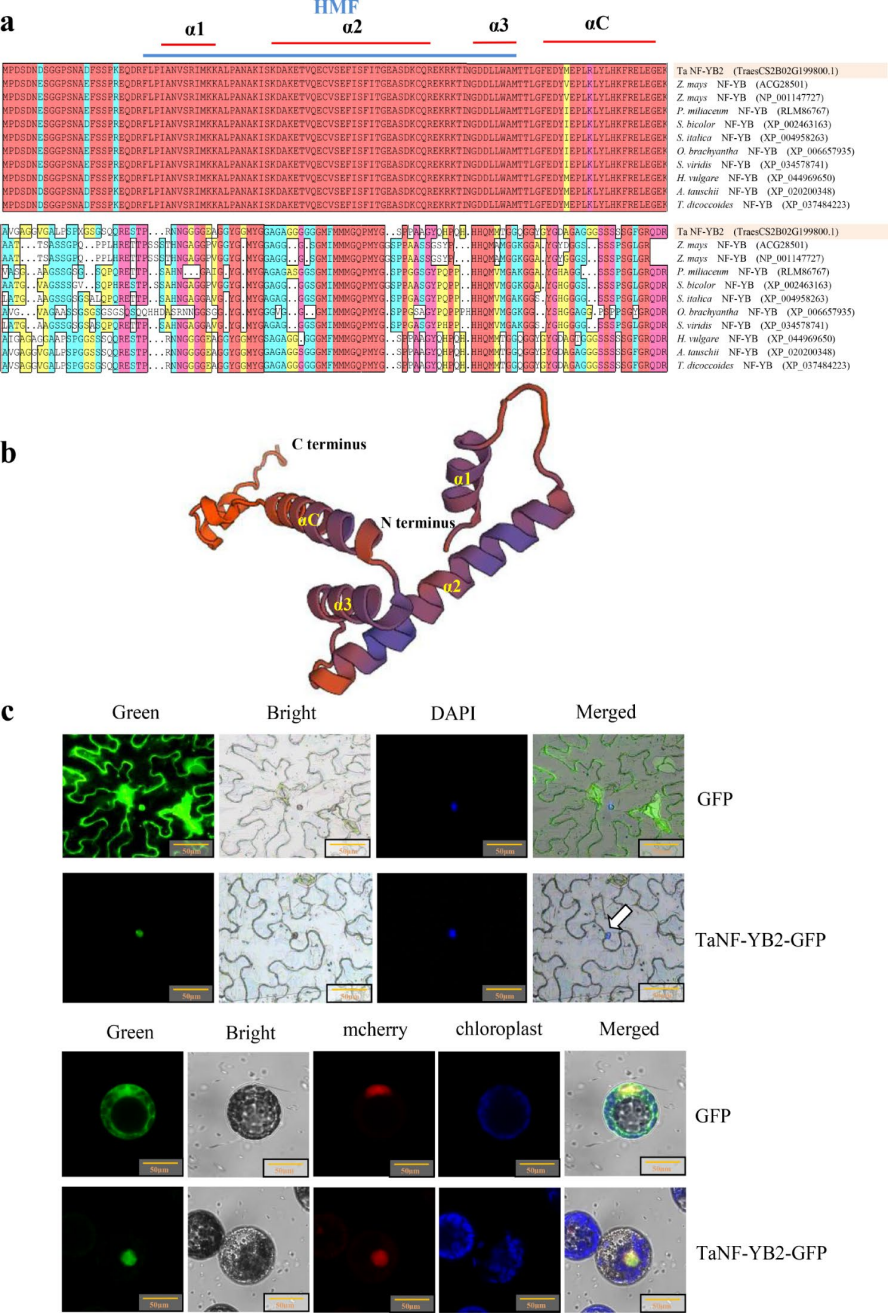
Characterization of the TaNF-YB2 protein. (**a**) Alignment results among the TaNF-YB2 protein and its counterparts across various plant species. (**b**) Simulated three-dimensional structure of the TaNF-YB2 protein. (**c**) Reporter GFP signals initiated from fusion TaNF-YB2-GFP or empty vector (GFP) of model *N. benthamiana* plants and wheat protoplasts. In (**a**) and (**b**), the conserved domains α1, α2, α3 and αC are highlighted. In (**c**), the arrows direct to nucleus

The NF-YB member *TaNF-YB2* (TraesCS2B02G199800.1) locates on chromosome 2B of *T. aestivum*, contains an open reading frame (ORF) of 678 bp in length that encodes a 225-aa polypeptide (Fig. S1). The predicted molecular mass and isoelectric point (pI) of TaNF-YB2 protein are 23.38 kDa and 5.74, respectively. *TaNF-YB2* shares high similarities to its homologous genes across diverse plant species at nucleic acid level, showing identities to the NF-YB genes in *Z. mays*, *E. sibiricus*, *T. dicoccoides*, *A. tauschii*, *H. vulgare*, and *S. bicolor* (Fig. S2). Amino acid sequence alignment revealed that TaNF-YB2 protein contains conserved domains specified by NF-YB members, including HMF (aa 25 - aa 89), α1 (aa 28–37 aa), α2 (aa 47 - aa 74), α3 (aa 82 - aa 89), and αC (aa 94 - aa 113) that are involved in binding CCAAT element and in interacting with proteins. This feature is similar to its plant counterparts in NF-YB family (Fig. 1a). Protein dimensional structure (3-D) prediction analysis on TaNF-YB2 defined its typical α-helical domain consisting of α1, α2, α3, αC (Fig. 1b). Subcellular localization experiment by detecting green fluorescent protein (GFP) signals initiated by fusion TaNF-YB2-GFP in transformed *N. benthamiana* epidermal cells and wheat protoplasts confirmed the nucleus targeting of TaNF-YB2 after endoplasmic reticulum (ER) assortment, contrasting to the free distribution of GFP signals in control group transformed by an empty vector (Fig. 1c). The nucleus location of TaNF-YB2 at subcellular level is consistent with its TF nature to regulate the transcription of downstream genes.

### Expression characterization of *TaNF-YB2* upon drought stress

**Fig. 2:**
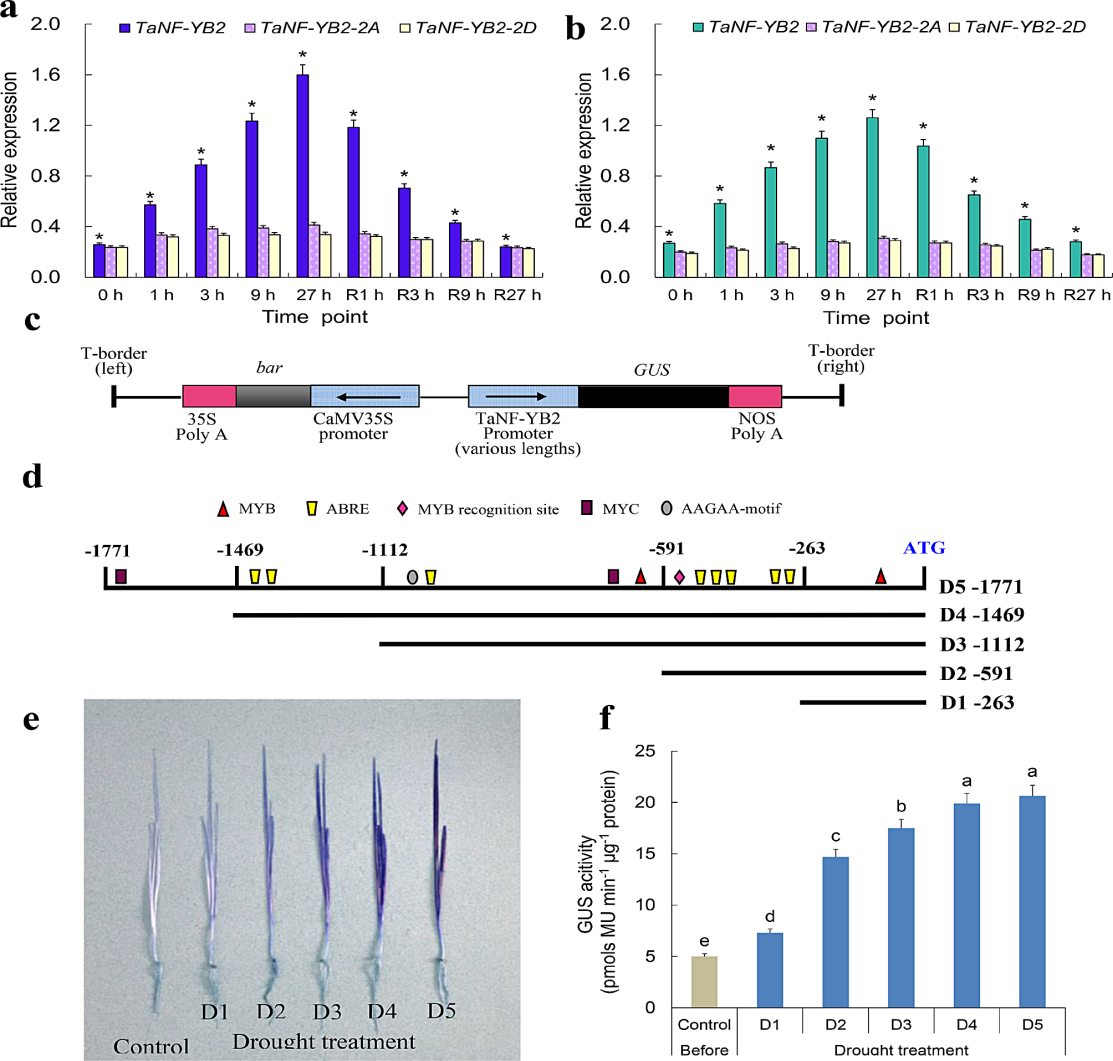
Expression patterns of *TaNF-YB2* and its homeologs in roots and leaves upon drought stress. (**a**) Expression patterns of *TaNF-YB2* and its homeologs in roots. (**b**) Expression patterns of *TaNF-YB2* and its homeologs in leaves. (**c**) Schematic representation of the binary expression cassettes integrated by *TaNF-YB2* promoters. (**d**) Diagrams for truncated *TaNF-YB2* promoter regions upstream the reporter gene *GUS*. (**e**) GUS histochemical staining results. (**f**) GUS activities. In (**a**-**b**), 1 h, 3 h, 9 h, and 27 h, time points after drought treatment. R1 h, R3 h, R9 h, and R27 h, time points during normal recovery treatment for the wheat seedlings challenged by 27 h drought stress. 0 h, time point prior to drought treatment. In (**f**), bars represent the SE from four independent assays and different letters indicate the significant difference (Student′s *t*-test, *P* < 0.05)

A 27 h regime of drought treatment and a normal recovery condition for wheat seedlings were used to address the expression patterns of *TaNF-YB2* and its homeologs (i.e., *TaNF-YB2-2A* and *TaNF-YB2-2D*). Upon drought signaling, the transcripts of *TaNF-YB2* in both root and aerial tissues were shown to be gradually elevated following the progression of drought condition, which reached a peak at end of the drought treatment (showing almost 6.0- and 4.7-fold increments in roots and leaves after 27 h drought treatment with respect to 0 h, respectively, Fig. 2a-b and Fig. S3). The homeologs of *TaNF-YB2*, *TaNF-YB2-2A* and *TaNF-YB2-2D*, also displayed enhanced expression in tissues treated by drought stress, but the induction effects of them upon drought were lower than *TaNF-YB2* (showing 1.8- to 1.6-fold increase on transcripts after 27 h drought treatment compared with 0 h, respectively, Fig. 2a-b). Upon a normal recovery condition, the transcripts of *TaNF-YB2* and its homeologs in both roots and leaves were all gradually restored following the progression of the recovery treatment, showing reduction on transcripts of them after a 27 h recovery treatment and restored similarly to those shown before drought treatment (0 h) (Fig. 2a-b). *TaNF-YB2* thus is suggested to be sensitive in drought response and possibly involved in mediating plant adaptation to drought stress.

Prediction analysis for *cis*-acting regulatory elements in *TaNF-YB2* promoter (2-kb in length upstream of translation start codon ATG) identified a subset of critical *cis*-acting regulatory elements involving gene transcription, gene expression efficiency regulation, and osmotic stress signaling response. For example, boxes TATA and CAAT that act as two of the conserved motifs involving interaction with RNA polymerase and regulation of gene transcription efficiency are located at positions close to ATG (Table S3), suggesting their potential roles in mediating transcription of *TaNF-YB2*. In addition, ABRE, recognition sites for transcription factors MYB, MBS, and MYC, the critical elements involving gene response to ostmotic stress signaling, are also situated at the *TaNF-YB2* promoter (Table S3). Therefore, these *cis*-acting regulatory elements are suggested to be involved in *TaNF-YB2* response upon drought stress.

To characterize the roles of the critical elements in mediating gene transcription efficiency and drought stress response, several truncated promoter fragments covering different elements/element amounts were subjected to construction of binary cassettes to drive expression of reporter gene *GUS* (Fig. 2c-d). Histochemical glucuronidase (GUS) staining analysis on the wheat leaves transformed above cassettes revealed that the samples harboring *TaNF-YB2*_pro263_-*GUS* (D1, Pro263), *TaNF-YB2*_pro591_-*GUS* (D2, Pro591), *TaNF-YB2*_pro1112_-*GUS* (D3, Pro1112), *TaNF-YB2*_pro1469_-*GUS* (D4, Pro1469), and *TaNF-YB2*_pro1771_-*GUS* (D5, Pro1771) displayed modified expression patterns of reporter gene under drought condition (Fig. 2e and Fig. S4). Compared with those shown before drought treatment, GUS activities for D1 to D5 were all increased, with gradual enhancement following the extension of promoter length (Fig. 2f). Therefore, the drought response element MYB motif (-88) in D1, ABRE and MYB recognition site (-323, -330, -333, -473, -487, -507, and − 544) in D2, MYB, MYC, ABRE, and AAGAA-motif (-657, -763, -1002, -1004, and − 1036) in D3, ABRE (-1351 and − 1395) in D4 and MYC (-1739) in D5, positively impact on the transcription regulation of *TaNF-YB2* upon drought signaling.

### TaNF-YB2 protein interacts with TaNF-YA7 and TaNF-YC7 to establish a heterotrimer

**Fig. 3:**
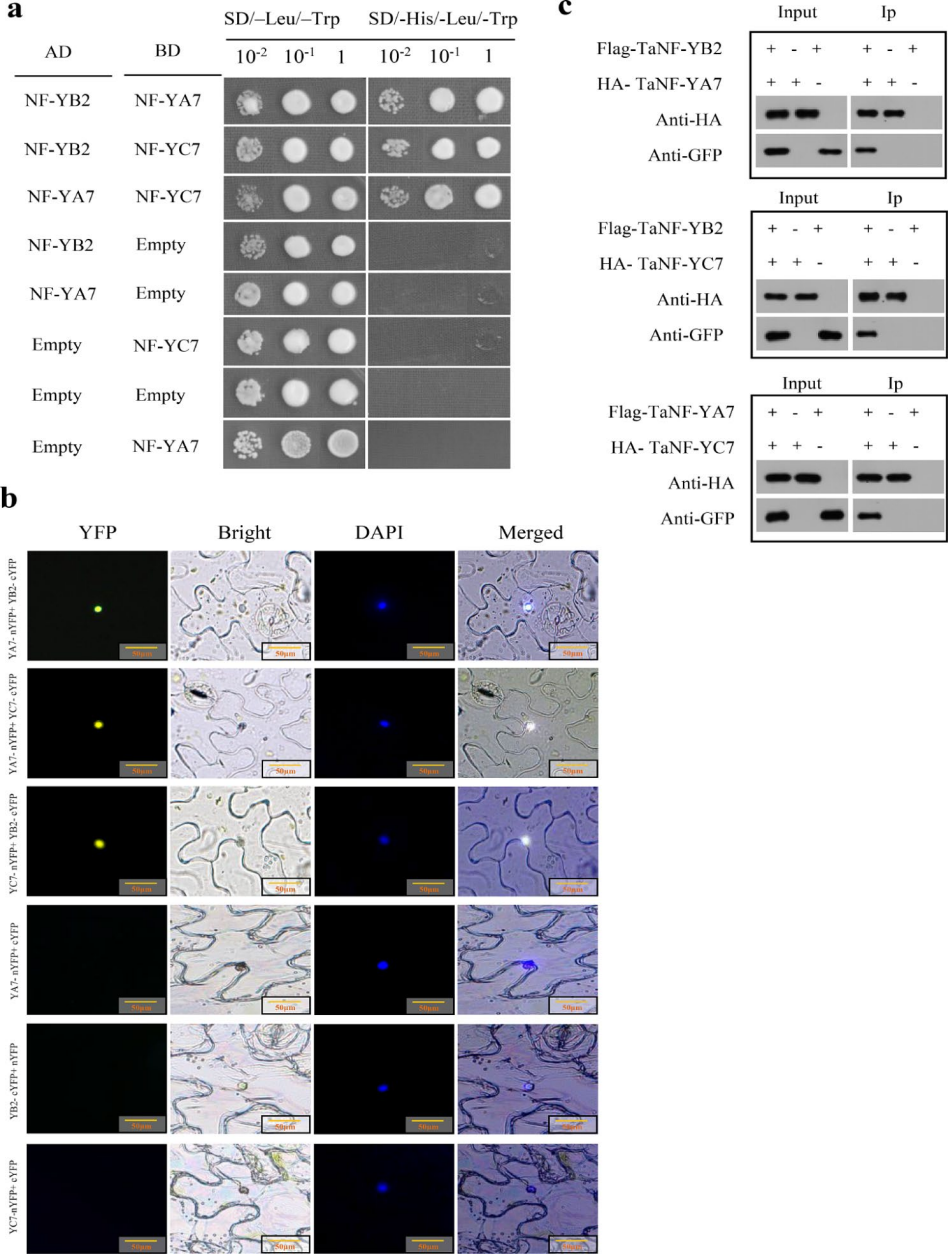
Protein interaction assay results among the TaNF-YB2 protein and the members in subfamilies NF-YA and NF-YC in *T. aestivum*. (**a**) Yeast two-hybrid results. (**b**) BiFC assay results. (**c**) Co-IP assay results

Protein-protein interaction characterizations on TaNF-YB2 were performed using Y-2 H, BiFC, and Co-IP to identify its partners in subfamilies NF-YA and NF-YC that involve constitution of a putative heteotrimer. Y-2 H analysis revealed that among three members in subfamily NF-YA (i.e., TaNF-YA2, TaNF-YA3, and TaNF-YA7), TaNF-YA7 was specifically interacted by TaNF-YB2 (Fig. 3a and Fig. S5). Similarly, among 8 members in subfamily NF-YC (i.e., TaNF-YC1 to TaNF-YC8), TaNF-YC7 was specifically interacted by the TaNF-YB2 protein (Fig. 3a and Fig. S6). Likewise, the two partners of TaNF-YB2, namely TaNF-YA7 and TaNF-YC7, interacted each other in the same assay system (Fig. 3a). These results from yeast two-hybrid assays indicated a heterotrimer established by TaNF-YB2, TaNF-YA7, and TaNF-YC7 (TaNF-YB2/TaNF-YA7/TaNF-YC7), which is involved in transcriotional regulation on downstream genes by synergistic action upon drought stress signaling.

BiFC and Co-IP assays on TaNF-YB2 and its interaction partners were conducted. The analysis results verified the interaction processes and cellular localizations of protein-protein interactions. Of which, BiFC assays indicated that strong nuclear yellow fluorescent protein (YFP) signals were identified in the nucleus of model plant *N. Benthamiana* co-transformed by cassettes TaNF-YB2-cYFP and TaNF-YA7-nYFP, TaNF-YB2-cYFP and TaNF-YC7-nYFP, and TaNF-YA7-nYFP and TaNF-YC7-cYFP (Fig. 3b). Likewise, Co-IP analyses confirmed above interaction processes obtained from the Y-2 H and BiFC assays (Fig. 3c). Collectively, the results from protein-protein interaction analysis verified that TaNF-YB2 involves constitution of a heterotrimer, namely TaNF-YB2/TaNF-YA7/TaNF-YC7, which functions as a transcription factor to modulate physiological stress responses in plants through regulating transcription of the downstream genes.

### *TaNF-YB2* positively regulates plant drought response

**Fig. 4:**
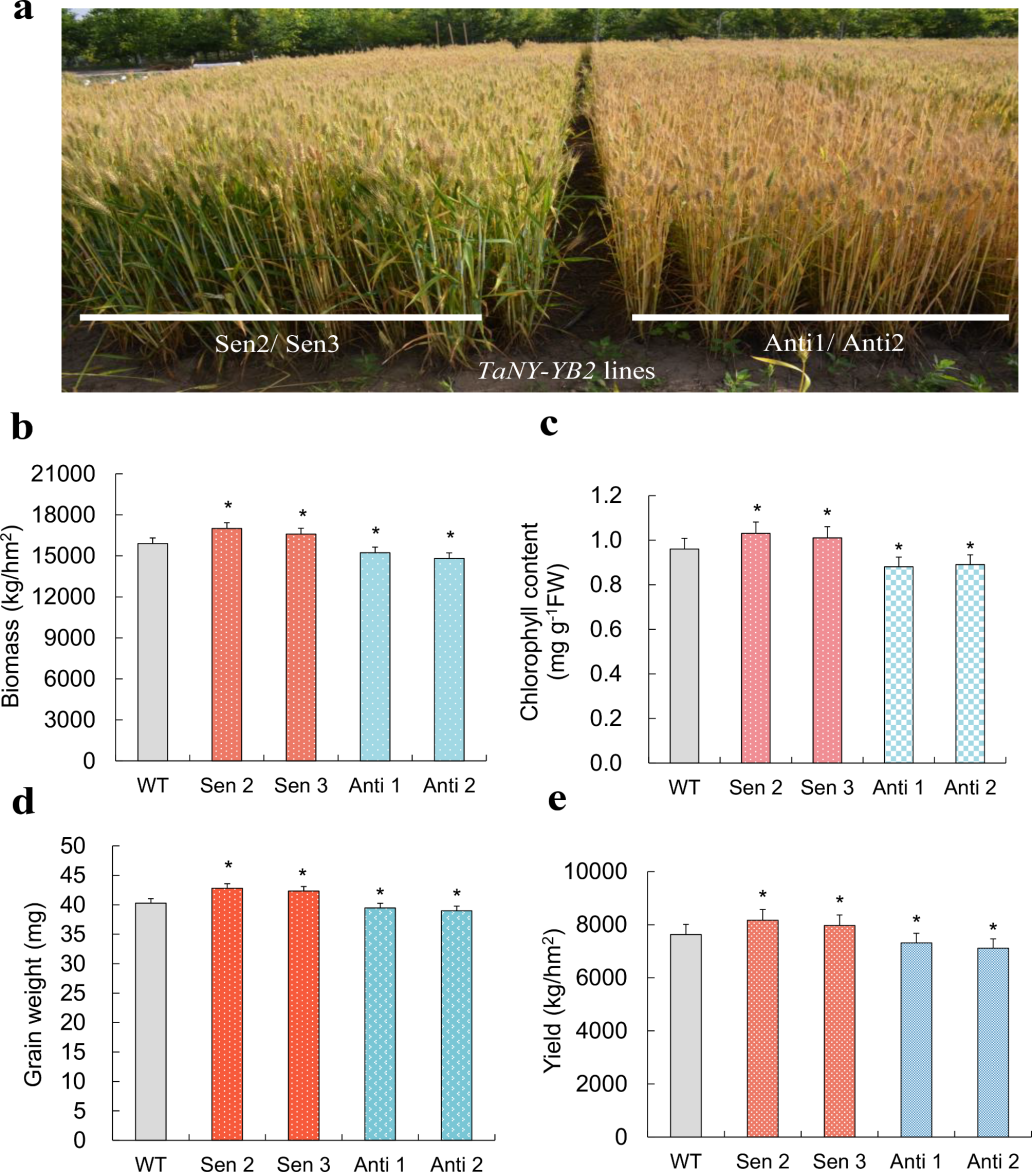
Phenotypes, growth and agronomic traits of the *TaNF-YB2* transgenic lines under normal condition and drought treatment. (**a**) Phenotypes of *TaNF-YB2* transgenic lines at seed filling stage. (**b**) Plant biomass of TaNF-YB2 transgenic lines at seed filling stage. (**c**) Chlorophyll contents in upper leaves of *TaNF-YB2* transgenic lines at seed filling stage. (**d**) Grain weights of *TaNF-YB2* transgenic lines at maturity. (**e**) Yields of *TaNF-YB2* transgenic lines at maturity. In (**b**-**e**) data presented are means ± standard deviation (*n* = 3). Student′s *t*-test was used to test the statistical significance (* *p* < 0.05) between WT and the transgenic lines under drought treatment

Transgenic wheat lines for *TaNF-YB2* with overexpression and knockdown expression were generated to characterize their roles in mediating plant drought responses (Fig. S7). Under field drought condition (water-saving management without irrigation during seed-filling stage), the lines with overexpression of *TaNF-YB2* (Sen 2 and Sen 3) displayed significantly improved plant growth and biomass (Fig. 4a-b and Fig. S8) and increased chlorophyll contents of upper leaves (Fig. 4c) at mid-seed filling stage, elevated grain weights (Fig. 4d), and enhanced yields (Fig. 4e) compared with wild type (WT). In contrast, the lines with knockdown of *TaNF-YB2* (Anti 1 and Anti 2) deteriorated plant growth and biomass (Fig. 4a-b), decreased chlorophyll contents of upper leaves (Fig. 4c) at mid-seed filling stage, lowered grain weights (Fig. 4d) and reduced yields (Fig. 4e) at maturity with respect to WT. These altered growth traits in the transgenic lines suggest that *TaNF-YB2* plays essential roles in modulating plant drought responses.

### *TaNF-YB2* modifies plant drought adaptation ascribes largely to its regulation on stomata movement, osmolyte accumulation, and ROS homeostasis

**Fig. 5:**
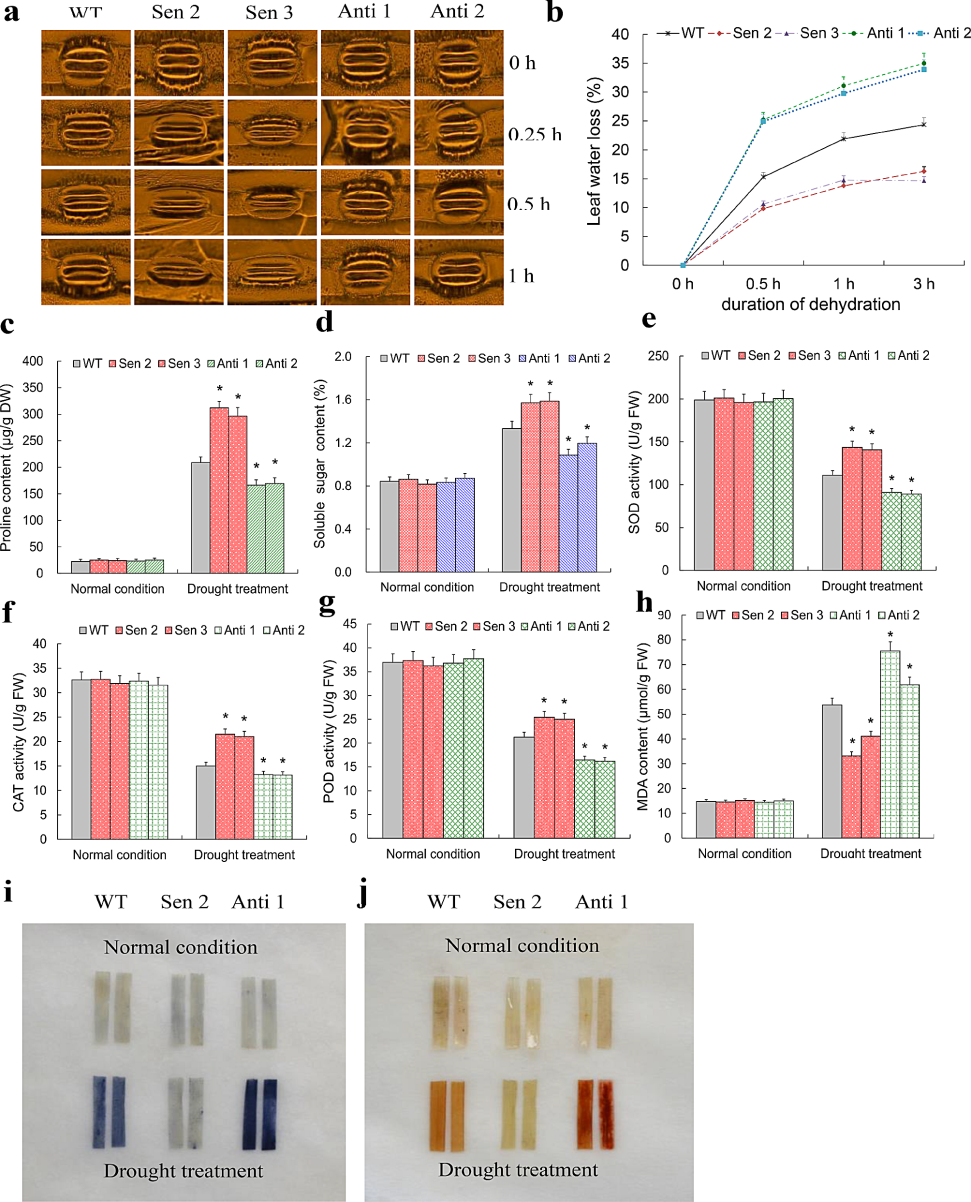
Osmotic stress-associated indices of the *TaNF-YB2* transgenic lines cultured under normal condition and drought treatment. (**a**) Stomata properties upon a 1 h-regime drought stress. (**b**) Water loss rate of the detached leaves following a 3 h-regime water deficit treatment. (**c**) Proline contents under normal condition and drought treatment. (**d**) Soluble sugar contents under normal condition and drought treatment. (**e**) SOD activities under normal condition and drought treatment. (**f**) CAT activities under normal condition and drought treatment. (**g**) POD activities under normal condition and drought treatment. (**h**) MDA contents under normal condition and drought treatment. (**i**) Histochemical staining for uperoxide anion using nitroblue tetrazolium (NBT) under normal condition and drought treatment. (**j**) Histochemical staining H_2_O_2_ using 3,3 diaminobenzidine (DAB). In (**b**-**h**) data presented are means ± standard deviation (*n* = 3). Student′s *t*-test was used to test the statistical significance (* *p* < 0.05) between WT and the transgenic lines under drought treatment

A suite of osmotic stress-associated physiological indices were assessed in the drought-challenged transgenic lines of *TaNF-YB2* using upper leaf samples at mid-seed filling stage (Fig. 5). Results showed that the stomata in all transgenic lines and WT were swiftly acclimated to closing along with drought stress duration (Fig. 5a). However, the lines with overexpression of *TaNF-YB2* exhibited much faster whereas those with knockdown of target gene slower on stomata closing than WT at indicated times during the treatment (i.e., 0.25, 0.5, and 1 h) (Fig. 5a). Results on leaf water losing rate (WLR) analysis on the drought-challenged transgenic lines and WT were in concordant with the stomata closing rates; the lines with *TaNF-YB2* overexpression were lower whereas the lines with above gene knockdown expression higher on leaf WLRs at various time points (0.5, 1, and 3 h) during a 3-h dehydration condition (Fig. 5b). Additionally, the lines with gene overexpression were much higher on contents of osmolytes, namely proline and soluble sugar (Fig. 5c-d), activities of antioxidant enzymes (AE), namely superoxde dismutase (SOD), catalase (CAT), and peroxidase (POD) (Fig. 5e-g), and lower contents of malondialdehyde (MDA), superoxide anion and hydrogen peroxide (H_2_O_2_) (Fig. 5h-j) under drought than WT at mid-filling stage. In contrast, the lines with knockdown expression of *TaNF-YB2* were much lower on contents of proline and soluble sugar (Fig. 5c-d), activities of SOD, CAT, and POD (Fig. 5e-g), and higher contents of MDA, superoxide anion and H_2_O_2_ (Fig. 5h-j) under drought than WT plants. These results on *TaNF-YB2*-mediated osmotic stress-associated physiological processes were consistent with the gene functions in positively regulating plant adaptation to drought stress.

### *TaNF-YB2* transriptionally regulates expression of distinct P5CS and AE family genes

**Fig. 6:**
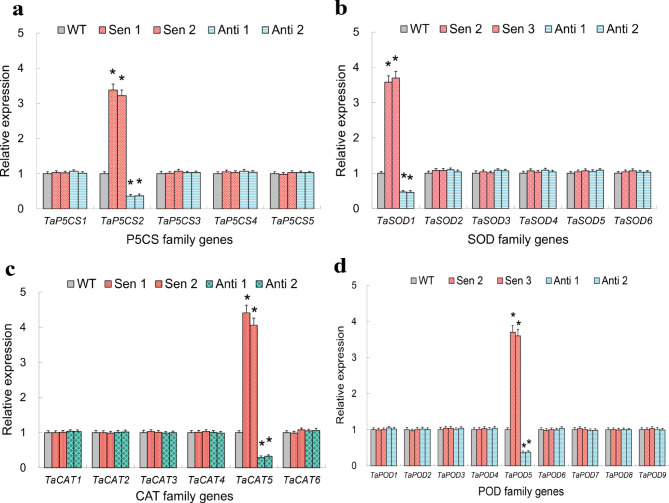
Expression patterns of the P5CS and AE family genes in the *TaNF-YB2* transgenic lines upon drought stress. (**a**) Expression patterns of P5CS genes. (**b**) Expression patterns of SOD genes. (**c**) Expression patterns of CAT genes. (**d**) Expression patterns of POD genes. In (**a**-**d**) data presented are means ± standard deviation (*n* = 3). Student′s *t*-test was used to test the statistical significance (* *p* < 0.05) between WT and the transgenic lines under drought treatment

Given that the significantly modified osmolyte contents and ROS-associated parameters were observed in the *TaNF-YB2* lines under drought conditions, the transcripts of a subset of genes in families of ^∆^1-pyrroline-5-carboxylate synthase (P5CS) and antioxidant enzyme (AE) (i.e., SOD, CAT, and POD encoding genes) that potentially modulate proline biosynthesis and cellular reactive oxygen species (ROS) homeostasis, respectively, were detected in the *TaNF-YB2* transgenic lines cultivated under field drought condition. Among the 5 P5CS genes (*TaP5CS1* to *TaP5CS5*), 6 SOD genes (*TaSOD1* to *TaSOD6*), 6 CAT genes (*TaCAT1* to *TaCAT6*), and 9 POD genes (*TaPOD1* to *TaPOD9*) examined, the P5CS family gene *TaP5CS2*, SOD family gene *TaSOD1*, CAT family gene *TaCAT5*, and POD family gene *TaPOD5* displayed modified expression in transgenic lines, all of which exhibited significantly upregulated in Sen 2 and Sen 3 and drastically downregulated transcripts in Anti 1 and Anti 2 compared with those shown in WT (Fig. 6a-d). These expression results suggested the putative functions of these differential P5CS and AE genes in modulating osmolyte (i.e., proline) and ROS homeostasis in the *TaNF-YB2* lines.

**Fig. 7:**
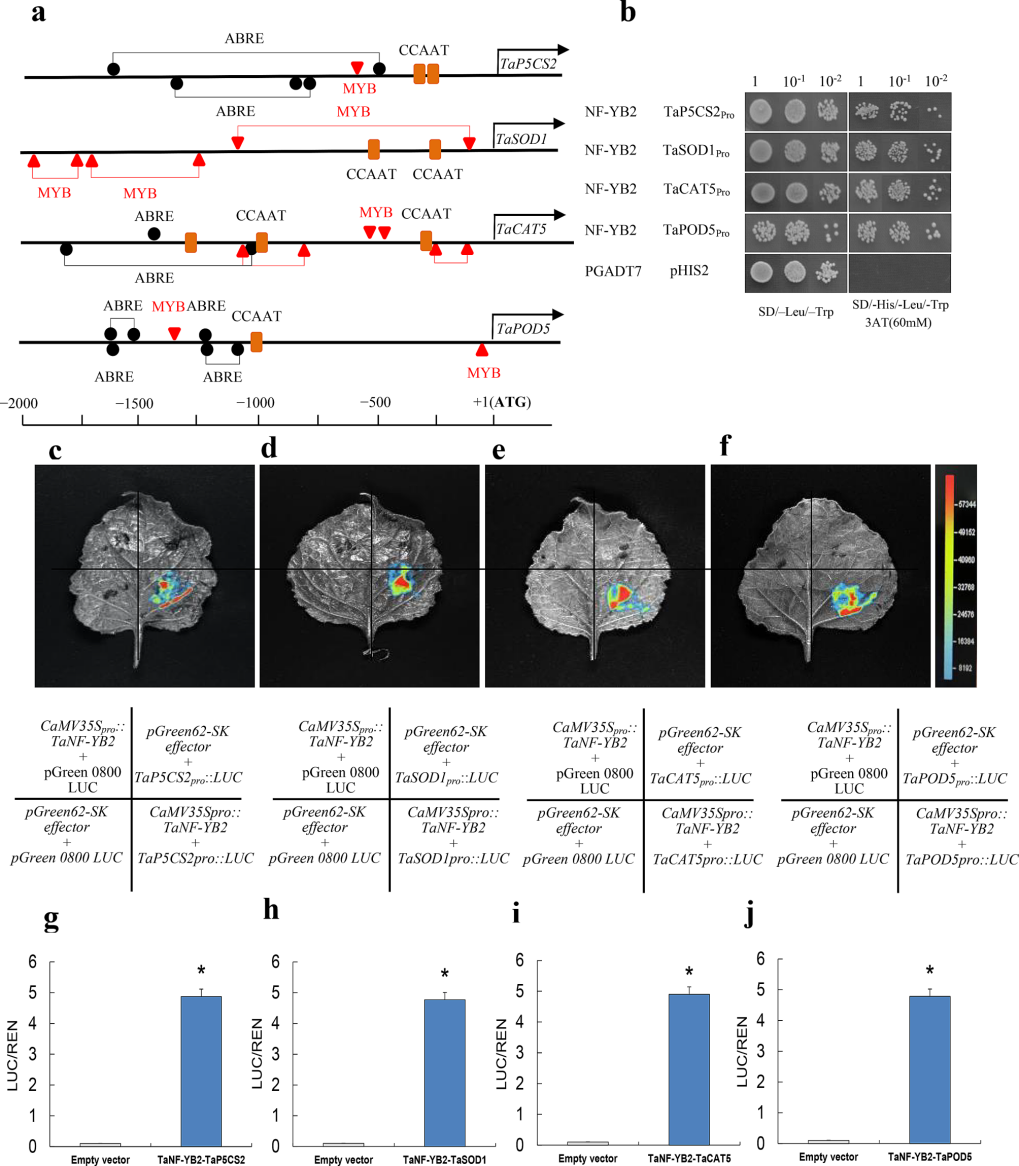
Transcriptional activation assay results on distinct osmotic stress-associated genes underlying TaNF-YB2 regulation. (**a**) Diagram showing locations of the osmotic stress-associated *cis*-acting elements in promoters of *TaP5CS2*, *TaSOD1*, *TaCAT5* and *TaPOD5*. (**b**) Yeast one-hybrid analysis of TaNF-YB2 showing its binding nature with promoters of stress genes *TaP5CS2*, *TaSOD1*, *TaCAT5*, and *TaPOD5*. (**c**-**f**) Fluorescein signals detected from *N. benthamiana* epidermal cells co-transformed with cassettes CaMV35S_pro_::*TaNF-YB2* and TaP5CS2_pro_::*LUC* (c), CaMV35S_pro_::*TaNF-YB2* and TaSOD1_pro_::*LUC* (d), CaMV35S_pro_::*TaNF-YB2* and TaCAT5_pro_::*LUC* (e), and CaMV35S_pro_::*TaNF-YB2* and TaPOD5_pro_::*LUC* (f). (g)-(j) LUC intensities derived from *N. benthamiana* epidermal cells co-transformed with cassettes CaMV35S_pro_::*TaNF-YB2* and TaP5CS2_pro_::*LUC* (g), CaMV35S_pro_::*TaNF-YB2* and TaSOD1_pro_::*LUC* (h), CaMV35S_pro_::*TaNF-YB2* and TaCAT5_pro_::*LUC* (i), and CaMV35S_pro_::*TaNF-YB2* and TaPOD5_pro_::*LUC* (j). In (a), ABRE (ACGTG, TACGGTC, GCCGCGTGGC, CGTACGTGCA, CACGTG) and MYB (CAACAG, CAACCA, TAACCA) are *cis*-regulatory elements situated in TaP5CS2, TaSOD1, TaCAT5, and TaPOD5 promoters. In (**g**-**j**) data presented are means ± standard deviation (*n* = 3). Student′s *t*-test was used to test the statistical significance (* *p* < 0.05) between epidermal cells transformed with empty vector and cassette combinations

Yeast one hybrid assays were conducted to define the protein/DNA binding property between TaNF-YB2 and the promoter regions of a set of stress response genes, namely *TaP5CS2*, *TaSOD1*, *TaCAT5*, and *TaPOD5*. Results showed that the yeast colonies co-transformed with pGADT7-*TaNF-YB2* and pHIS2-TaP5CS2_Pro_, pGADT7-*TaNF-YB2* and pHIS2-TaSOD1_Pro_, and pGADT7-*TaNF-YB2* and pHIS2-TaCAT5_Pro_, pGADT7-*TaNF-YB2* and pHIS2-TaPOD5_Pro_ grew normally in both doubled-deficient medium and triple-deficient medium with 60 mM 3-AT (Fig. 7b). These findings verified that TaNF-YB2 possesses abilities in binding with the promoter regions of *TaP5CS2*, *TaSOD1*, *TaCAT5*, and *TaPOD5* (Fig. 7a-b).

The stress responsive genes (*TaP5CS2*, *TaSOD1*, *TaCAT5* and *TaPOD5*) that were differentially expressed in the drought-challenged *TaNF-YB2* transgenic lines were subjected to transcriptional activation assays to define their modulation underlying the wheat NF-YB TF at transcriptional level. Prediction analysis revealed that a suite of *cis*-acting regulatory elements, namely those involved in regulating gene response to osmotic stresses including ABRE and MYB TF recognition sites are situated at the promoter regions of them (Fig. 7a; Table S4). Further transcriptional assays indicated that in model plant *N. benthamiana* system, clear fluorescein signals were detected in the epidermal cells of the model plant co-transformed with the cassette combinations, namely CaMV35S_pro_::*TaNF-YB2* and TaP5CS2_pro_::*LUC*, CaMV35S_pro_::*TaNF-YB2* and TaSOD1_pro_::*LUC*, CaMV35S_pro_::*TaNF-YB2* and TaCAT5_pro_::*LUC*, and CaMV35S_pro_::*TaNF-YB2* and TaPOD5_pro_::*LUC* (Fig. 7c-j). In addition, under the condition to be co-existence of TaNF-YB2, TaNF-YA7, and TaNF-YC7, enhanced fluorescence intensity was observed in the transformed *N. benthamiana* epidermal cells, indicating that these TF proteins function high efficiency in regulating downstream target transcription using a heterotrimer mode after specific interaction with the binding motif of stress response gene promoters (Fig. S10).

**Fig. 8:**
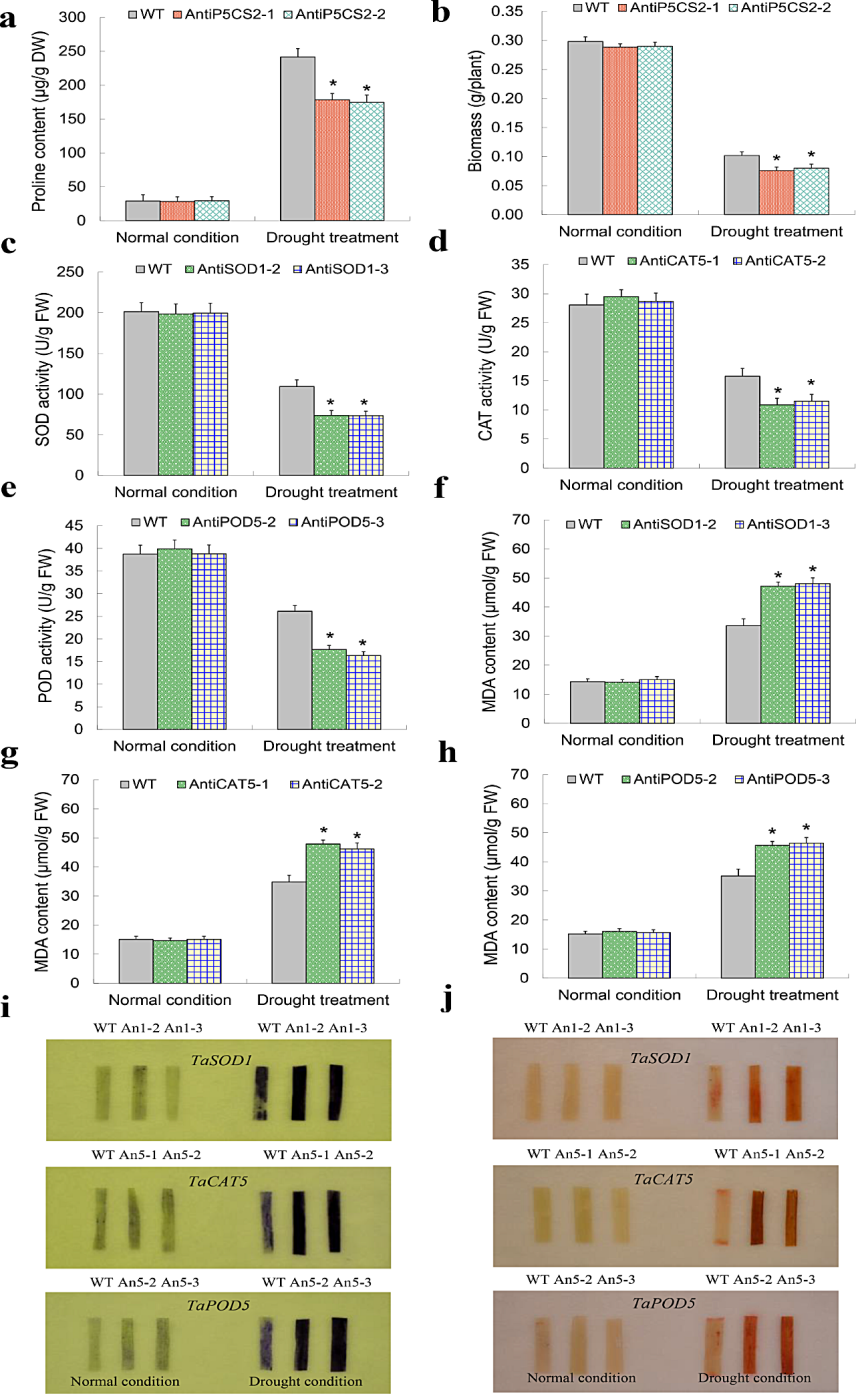
Functional characterizations of the osmotic stress-associated genes under drought condition. (**a**) Proline contents in *TaP5CS2* knockdown lines. (**b**) Plant biomass in *TaP5CS2* knockdown lines. (**c**) SOD activities in *TaSOD1* knockdown lines. (**d**) CAT activities in *TaCAT5* knockdown lines. (**e**) POD activities in *TaPOD5* knockdown lines. (**f**-**h**) MDA contents in lines with knockdown expression of *TaSOD1* (**f**), *TaCAT5* (**g**), and *TaPOD5* (h). (**i**) Histochemical staining for uperoxide anion using nitroblue tetrazolium (NBT) under normal condition and drought treatment. (**j**) Histochemical staining H_2_O_2_ using 3,3 diaminobenzidine (DAB). In (i)**-**(j) An2-1 and An2-2 correspond to AntiP5CS2-1 and AntiP5CS2-2, An1-2 and An1-3 correspond to AntiSOD1-2 and TaSOD1-3, An5-1 and An5-2 correspond to AntiCAT5-1 and TaCAT5-2, and An5-2 and An5-3 correspond to AntiPOD5-2 and TaPOD5-3. In (**a-h**) data presented are means ± standard deviation (*n* = 3). Student′s *t*-test was used to test the statistical significance (* *p* < 0.05) between WT and the transgenic lines under drought treatment

Transgene analyses on these stress-defensive genes were further conducted to verify their biological functions. As expected, compared with WT, TaP5CS2-1 and TaP5CS2-2, two lines with knockdown expression of *TaP5CS2* deteriorated drastically decreased plant proline contents and biomass (Fig. 8a-b); TaSOD1-2 and TaSOD1-3, two lines with repression of *TaSOD1* lowered plant SOD activities (Fig. 8c); TaCAT5-1 and TaCAT5-2, two lines with knockdown of *TaCAT5* decreased plant CAT activities (Fig. 8d); and TaPOD5-2 and TaPOD5-3, two lines with repressed *TaPOD5* expression reduced plant POD activities (Fig. 8e). Moreover, all of the lines with knockdown expression of AE genes increased plant MDA contents relative to WT plants (Fig. 8f-h) and displayed more accumulation of superoxide anions (Fig. 8i) and H_2_O_2_ (Fig. 8j). Therefore, distinct P5CS and AE genes (i.e., *TaP5CS2*, *TaSOD1*, *TaCAT5*, and *TaPOD5*) are suggested to be controlled underlying *TaNF-YB2* to contribute plant drought response by enhancing osmolyte accumulation and improving cellular ROS scavenging.

### Transcripts abundance of *TaNF-YB2* and its downstream stress-defensive genes highly correlates plant yield in wheat cultivars under field drought conditions

**Fig. 9:**
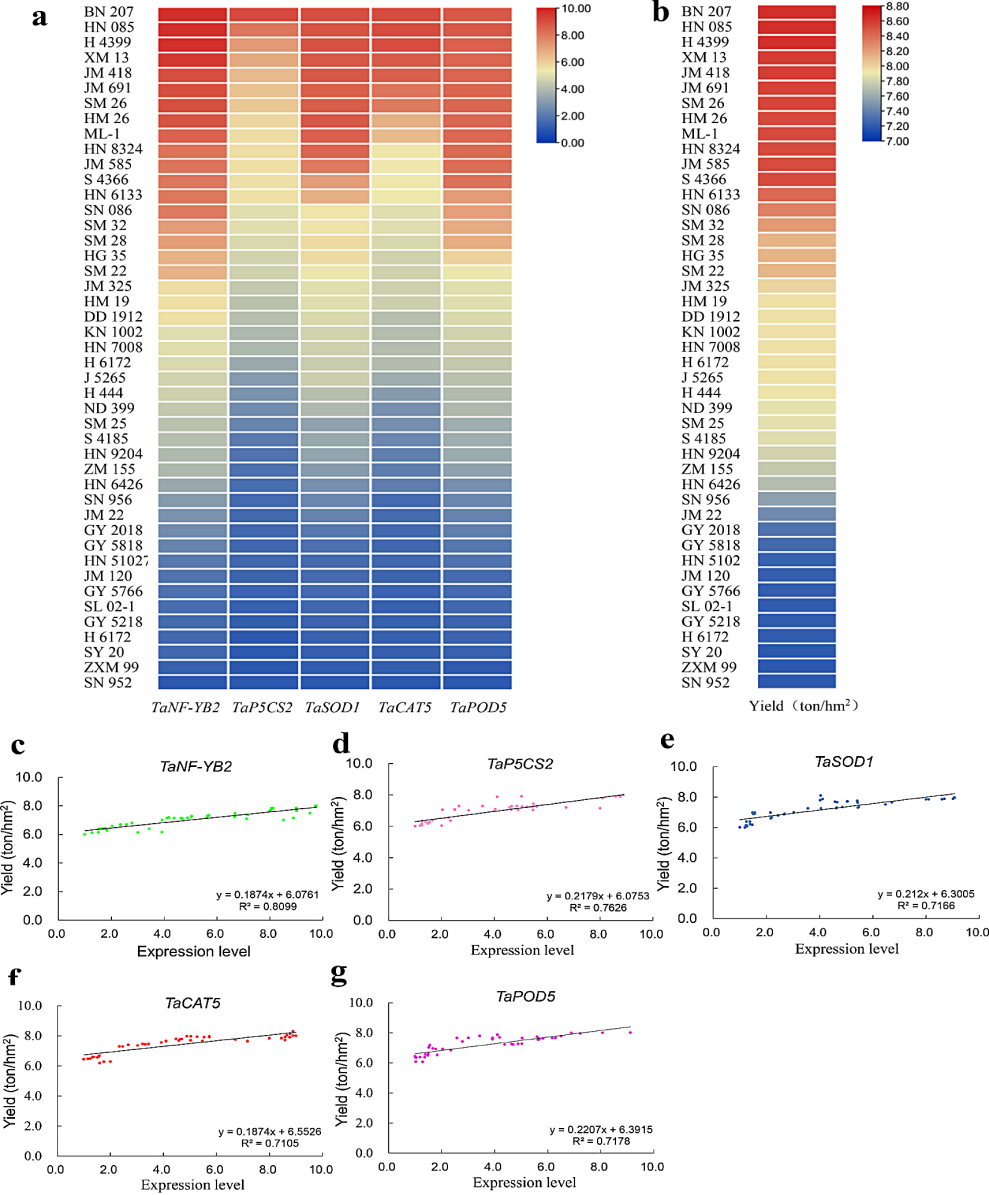
Characterizations on transcripts of *TaNF-YB2* and stress defensive genes and yields in a cultivation population under field drought condition. (**a**) Heat maps showing expression levels of *TaNF-YB2*, *TaP5CS2*, *TaSOD1*, *TaCAT5* and *TaPOD5* in tested wheat cultivars. (**b**) Heat maps showing yields in tested wheat cultivars. (**c**-**g**) Regression analysis results between the yield and transcripts of *TaNF-YB2* (**c**), yield and transcripts of *TaP5CS2* (**d**), yield and transcripts of *TaSOD1* (**e**), yield and transcripts of *TaCAT5* (**f**), and yield and transcripts of *TaPOD5* (**g**). In (**a**-**b**) the 45 tested wheat cultivars are shown at left positions and simplified based on registration names

The expression levels of *TaNF-YB2* and stress-responsive genes (*TaP5CS2*, *TaSOD1*, *TaCAT5*, and *TaPOD5*) were examined in a panel of 45 wheat cultivars that differed in drought tolerance in terms of plant yield behavior. The transcripts of these genes in upper leaves during the mid-filling stage exhibited significant variation under field drought conditions (Fig. 9a). Likewise, drastic difference on yield was observed at maturity under field drought condition across the wheat cultivars examined (Fig. 9b). Regression analysis was performed to characterize the relations among the gene transcripts and yield in the drought-challenged wheat cultivars. Results revealed significantly positive correlations between yield and the expression levels of *TaNF-YB2* and the four stress-responsive genes (Fig. 9c-g). These findings suggest that *TaNF-YB2* and distinct stress-defensive genes act synergistically at the transcriptional level to modulate plant drought response and contribute to plant drought adaptation capacity.

### Haplotype variation behaviors of *TaNF-YB2*

**Fig. 10:**
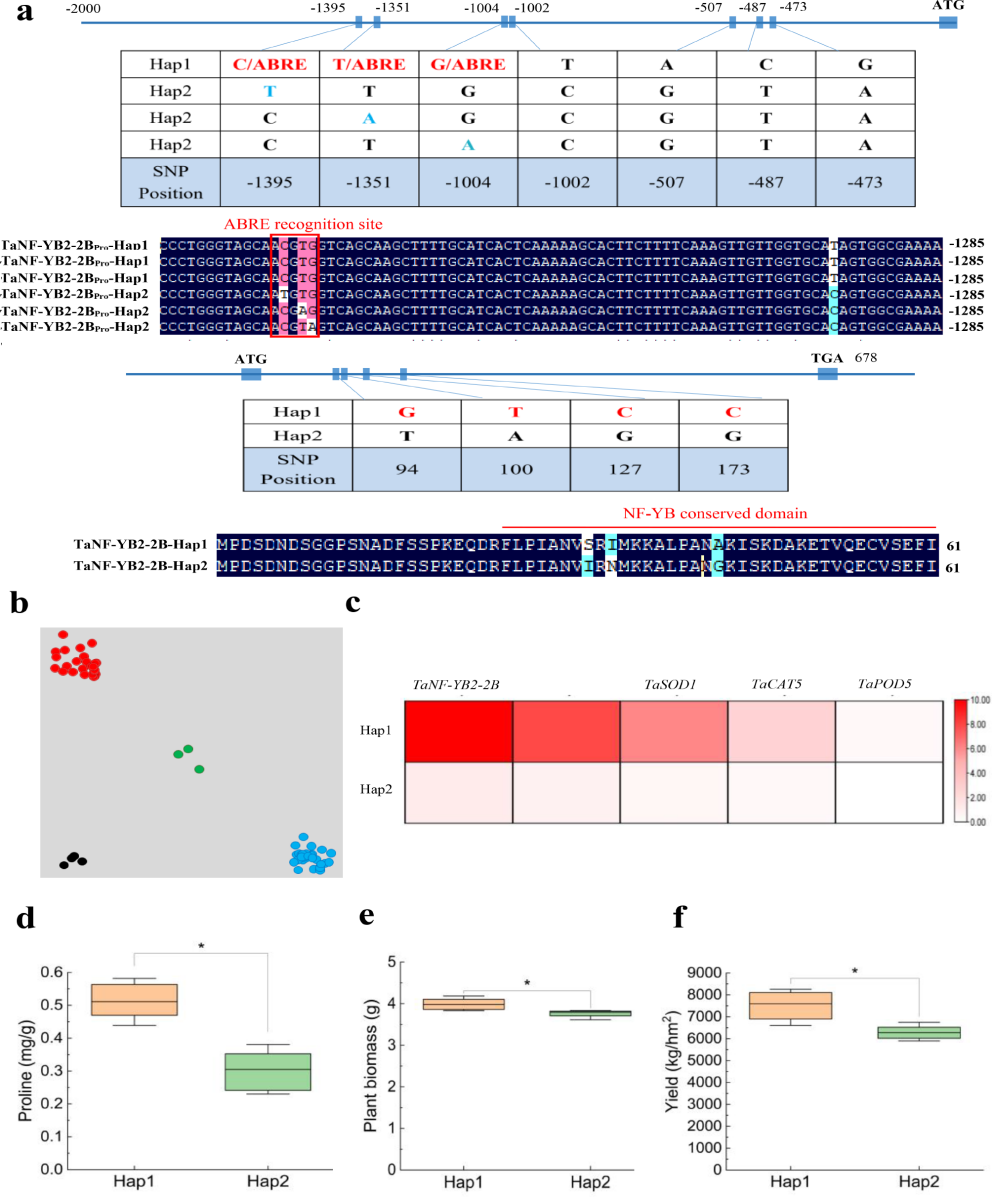
Effects of *TaNF-YB2* haplotypes on proline, plant biomass and yield traits.(**a**) Major *TaNF-YB2* haplotypes, Hap1 and Hap2, and the alignment of their deduced amino acid sequences. The polymorphic site shown in red was used to develop a haplotype-specific marker for *TaNF-YB2*. (**b**) Genotyping display of a panel of wheat cultivars using the haplotype-specific marker for *TaNF-YB2*. KASP, kompetitive allele-specific PCR. (**c**) Haplotypes heatmap clustering based on genes related to *TaNF-YB2*. Comparisons of proline (**d**), plant biomass (**e**), Yield (**f**) between the cultivars with contrasting haplotypes Hap1 and Hap 2

To identify sequence variants of *TaNF-YB2*, polymorphic sites in open reading frames (ORF) and flanking regions (i.e., 2-kb upstream of the start codon) were detected by sequencing the ORFs and gene promoter regions in the 45 wheat variety panel. 4 SNPs in ORF and 7 SNPs in promoter regions of *TaNF-YB2* formed two major haplotypes, namely TaNF-YB2-Hap1 and TaNF-YB2-Hap2 (Fig. 10a). Based on above results, a KASP marker referred to as TaNF-YB2-KASPA523T, was developed and used to genotype a panel of 45 elite wheat cultivars examined (Fig. 10b). The expression levels of *TaNF-YB2* and stress response genes (*TaP5CS2*, *TaSOD1*, *TaCAT5*, and *TaPOD5*) were characterized. Heatmap analysis revealed that wheat cultivars categorized into TaNF-YB2-Hap1 displayed higher expression levels than those into TaNF-YB2-Hap2 (Fig. 10c), conferring plants elevated proline content, plant biomass, and yield (Fig. 10d-f). These results together suggested that TaNF-YB2-Hap1 has been subjected to the positive selection in the modern wheat breeding programs.

## Discussion

### *TaNF-YB2* responses to drought stress at transcriptional level are associated with a suite of *cis*-elements situated in its promoter

Members of nuclear factor-Y (NF-Y), such as those encoding the NF-YB proteins have been documented to be extensively involved in plant responses to various environmental cues, such as osmotic stresses at transcriptional level. For example, a subset of the NF-Y genes across various plant species, including *(A) thaliana* [34], *(B) distachyon* [35], *B. napus* [36], *S. italica* [37], *G. max* [13], *P. vulgaris* [38], and *S. lycopersicum* [39], showed expression patterns to be tissue-specific and stress responsive. In this study, our expression analysis on *TaNF-YB2* by detecting its transcripts in tissues confirmed that it is response to the drought signaling. Previous investigations on gene transcription have suggested that the essential roles of distinct *cis*-acting regulatory elements situated at promoter in impacting on gene transcriptional response to stress signaling [40]. Based on an online search analysis, we identified a suite of elements related to gene responses to osmotic signaling, such as ABRE (motifs GACACGTGGC, ACGTG, GCAACGTGTC, CGCACGTGTC), AAGAA-motif (GAAAGAA), and recognition sites for transcription factors MYB (CAACCA, CCGTTG), and MYC (CATTTG), are situated at the *TaNF-YB2* promoter aside from boxes TATA and CAAT, which involve protein/DNA binding and transcriptional efficiency regulation. Higher frequencies of these *cis*-elements identified in *TaNF-YB2* promoter, namely, 10 for ABRE (-323, -330, -333, -473, -487, -507, -1002, -1004, -1351, -1395), 1 for AAGAA-motif (-1036), 2 for each recognition site MYB (-88, -657) and MYC (-763, -1739) suggested their putative functions on the gene response to drought stress. Moreover, our experiments on reporter GUS staining assay under control of a series of truncated *TaNF-YB2* promoter regions confirmed the roles of these *cis*-elements in intensifying gene transcriptional efficiency under drought stress. Therefore, the modified response of *TaNF-YB2* to drought stress is associated with distinct *cis*-acting elements in promoter which regulate gene transcription efficiency under stress conditions.

### TaNF-YB2 protein involves constitution of a heterotrimer with its NF-YA and NF-YC partners

NF-Y factor exerts biological functions in plants with a heterotrimer form that is comprised by three kinds of subunits, namely, NF-YA, NF-YB, and NF-YC [11]. Thus far, the proteins involving constitution of a distinct heterotrimer complex that impacts plant growth and development have been defined [15]. For example, a subunit NF-YC in Arabidopsis interacts with a NF-YB protein to establish a heterotrimer together with CONSTANS protein, playing a critical role in controlling plant flowering behavior [41]. In soybean, three NF-Y subunits including GmNF-YC14, GmNF-YB2, and GmNF-YA16 interact each other to be involved in plant osmotic stress tolerance through an ABA signaling pathway [42]. Subunits NF-YA in citrus plants referred to as CsNF-YA3 and CsNF-YA5 specifically interact with NF-YB subunits CsNF-YB6, CsNF-YB7, and CsNF-YB9, and NF-YC subunit CsNF-YC5, constituting a set of heterotrimer combinations among them to modulate plant growth and stress responses [43]. Likewise, CsNF-YA2, another member encoding subunit NF-YA exhibits the ability to establish hetorotrimers with CsNF-YB5/11 and CsNF-YC2/3, exerting roles in regulating transcriptional efficiencies of downstream genes [43]. In this study, to characterize the TaNF-YB2-involved constitution of a putative heterotrimer, we performed three kinds of protein-protein analysis approaches including Y-2 H, BiFC, and Co-IP assays. Results indicated that TaNF-YB2 specifically interacts with TaNF-YA7 and TaNF-YC7, two of the members in NF-YA and NF-YC families, respectively. Additionally, the interactions among them occur at nucleus based on detection of the reporter YFP signals in leaves of model plant *N. benthamiana* and wheat protoplasts co-transformed by gene-*YFP* fusions. Therefore, the induced transcription/translation of *TaNF-YB2* upon drought signaling mediates plant osmotic stress response through a TaNF-YB2-invloved heterotrimer that exert roles in modulating transcription of downstream genes.

### *TaNF-YB2* confers plant drought adaptation by modulating stress-responsive physiological indices

NF-YB transcription factors play an important role in mediating plant resistance to abiotic stress by regulating downstream gene transcription [11, 42]. A number of studies have been conducted for characterizing the biological roles of NF-Y family genes in mediating plant responses to various abiotic stresses. Characterization on *PdNF-YB7*, a gene of the NF-YB family in popular that is upregulated in expression upon drought stress and ABA signaling, revealed that its overexpression confers plants an significant enhancement on drought resistance and yield formation capacity [20], which is associated with the gene function in promoting root system architecture (RSA) establishment, photosynthetic function, and water use efficiency (WUE) of plants under drought treatments [20]. Similarly, *GmNF-YB17* in soybean, a NF-YB family member being inducible under osmotic stress conditions, is involved in plant tolerance to drought stress; the transgenic lines overexpressing *GmNFYB17* displayed enhanced water retention capacity and improved productivity of plants once treated by dehydration stressor [44]. Modified expression of *NF-YB2* led to altered drought adaptation capacities on plants, with stronger and weaker ones in the lines overexpressing and knockdown expression of target gene, respectively [45]. In this study, we performed transgene analysis on *TaNF-YB2* to define its function in regulating plant drought responses. Significant variations on growth traits (i.e., phenotypes, plant biomass, and yield traits) and osmotic stress-associated physiological parameters (i.e., stomata closing rates (SCR), water losing rates (WLR), osmolytes contents, and cellular ROS-associated traits) were found in the lines with overexpression or knockdown of *TaNF-YB2* under drought treatment. Improvement on these physiological parameters in *TaNF-YB2*-overexpressing lines verified the gene functions in synergistically regulating plant adaptation to drought stress.

Previously, it was documented that promotion of stomata closing rate upon drought signaling [23], and improvement of osmolytes accumulation and cellular ROS homeostasis acts as critical factors in improving plant tolerance to osmotic stresses [46]. In this study, given the findings that *TaNF-YB2*-meidated plant drought tolerance is associated closely with its roles in promoting stomata closure, enhancing osmolytes accumulation, and improving ROS scavenging capacity, we investigated the molecular processes underlying *TaNF-YB2*-mediated drought adaptation based on expression and functional analyses on the P5CS and antioxidant enzyme (AE) family genes. We identified a suite of genes in above families mentioned, including *TaP5CS2*, a member in P5CS family and *TaSOD1*, *TaCAT5*, and *TaPOD5*, three genes in SOD, CAT, and POD families, were modified on transcription significantly in the *TaNF-YB2* transgenic lines under drought treatment with respect to WT plants. Our findings suggested that these genes are regulated underlying TaNF-YB2 to potentially impact on plant drought response.

Previous studies have shown that NF-YB members mediate gene stress response in plants using specific ABRE *cis*-acting elements (ACGTG/TC) that enhance transcription of stress-responsive genes [47]. In addition, some literatures record that transcription factors regulate a series of gene expression related to biotic or abiotic stress through interaction with MYB *cis*-acting elements (CAACCA/AG) on downstream gene promoters. Our analysis also identified a suite of ABRE and MYB binding elements located at the promoters of the stress-responsive genes, namely *TaP5CS2*, *TaSOD1*, *TaCAT5*, and *TaPOD5*. To further confirm the relationship between TaNF-YB2 and the stress-responsive genes, we conducted transcriptional activation assays using a model plant *N. benthamiana* system. Our results showed that TaNF-YB2 effectively activates the transcription efficiencies of these stress-responsive genes. Moreover, using the knockdown expression lines, we verified the biological functions of them in impacting the related growth and physiological traits under drought treatments. In conclusion, a suite of functional modules underlying TaNF-YB2, namely TaNF-YB2-*TaP5CS2*, TaNF-YB2-*TaSOD1*,TaNF-YB2-*TaCAT5* and TaNF-YB2-*TaPOD5* act synergistically to modulate plant drought response. Further characterization of the transcriptional mechanisms of these genes can deepen understanding of the physiology of plants under drought signaling in *T. aestivum*.

### Modified expression of *TaNF-YB2* and stress-responsive genes contribute to varied drought tolerance for wheat cultivars

Genetic variation is considered a fundamental element for enhancing crop quantitative traits, including yield components and drought tolerance. Various methods have been employed to enlarge the genetic variation in crop plants, such as introducing existing varieties, developing segregating materials through local or international nurseries, hybridization, and mutation breeding [48]. In addition, using parental lines with divergent genetic backgrounds, including unrelated and complementary genetic resources possessing suitable drought-adaptive and yield-enhancing traits, can help create superior breeding populations [48]. In this study, we assessed the expression levels of *TaNF-YB2* and the stress-responsive genes, namely *TaP5CS2*, *TaSOD1*, *TaCAT5* and *TaPOD5*, in a core variety panel consisting of 45 wheat cultivars with contrasting plant drought responses. Significant variations in expression levels of these genes were observed in flag leaves among the cultivars at mid-seed filling stage in the field experiment under water-saving irrigation management. Moreover, highly positive correlations were found between plant yield and the expression levels of *TaNF-YB2*, *TaP5CS2*, *TaSOD1*, *TaCAT5* and *TaPOD5*. Taken together, these findings indicate that the genes *TaNF-YB2*, *TaP5CS2*, *TaSOD1*, *TaCAT5* and *TaPOD5* play a crucial role in regulating plant yield production. Our findings indicated that the transcript abundance of the *TaNF-YB2*-consituted signaling module could be used as effective indices in evaluating the drought adaptation ability across wheat cultivars cultivated under drought conditions.

Based on characterizations on SNP behaviors for *TaNF-YB2* promoter in the wheat variety panel, we revealed the base variations in the sequence region flanking the ABRE-binding sites in promoter among the contrast drought-tolerant wheat cultivars. Two types of haplotype including TaNF-YB2-Hap1 and TaNF-YB2-Hap2 were suggested to impact plant drought response through affecting transcripts abundance of target gene. The haplotype TaNF-YB2-Hap1 conferred improved drought adaptation to the wheat cultivars, suggesting that this haplotype type could be one valuable target in the efforts for molecular breeding drought-tolerant cultivars in *T. aestivum*.

**Fig. 11:**
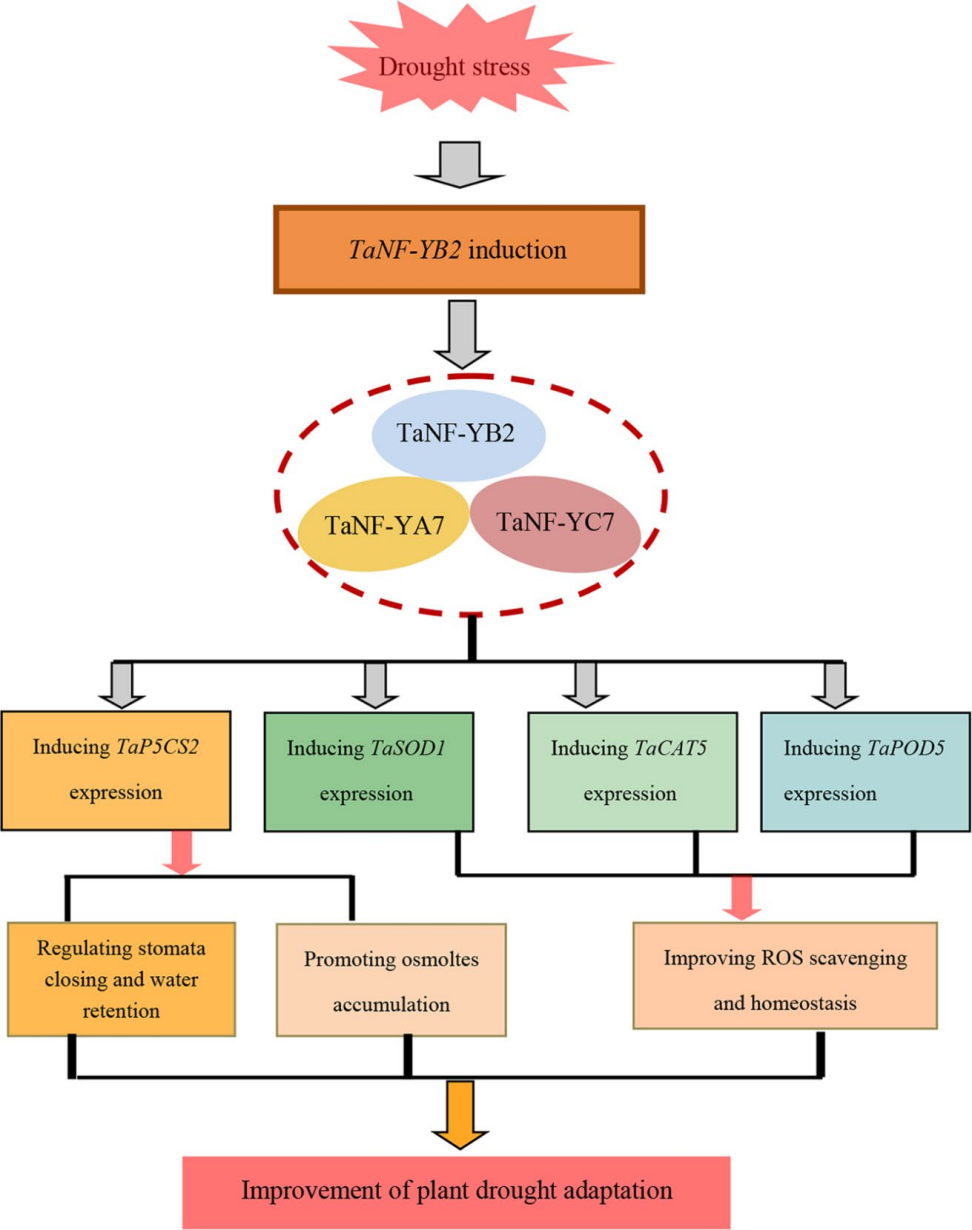
A working model for *TaNF-YB2* and its downstream partners in modulating plant adaptation to drought stress

Based on our investigations, we established a working model for *TaNF-YB2* and its downstream partners in mediating plant drought response (Fig. 11). Upon drought stress, the *TaNF-YB2* transcription is activated, whose translated product after induction acclimatizes to specifically interact with its partners in NF-YA and NF-YC subfamilies, namely, TaNF-YA7 and TaNF-YC7, to constitute a heterotrimer TaNF-YA7/TaNF-YB2/TaNF-YC7. The NF-Y TF then transcriptinally activates a set of osmotic stress-defensive genes in *T. aestivum*, namely a P5CS family member *TaP5CS2*, a superoxide dismutase family gene *TaSOD1*, a catalse family member *TaCAT5*, and a peroxidase family gene *TaPOD5*, which are upregulated in expression underlying TaNF-YB2 modulation to regulate physiological and molecular processes, including stomata response, water retention capacity of plants, osmolytes accumulation, and ROS homeostasis. These physiological processes underlying regulation of TaNF-YB2 are comprehensively integrated that contribute to plant adaptation to drought stress.

## Conclusions

Wheat NF-YB member *TaNF-YB2* is sensitive in response to drought stress; GUS histochemical staining analysis for truncated *TaNF-YB2* promoter regions confirmed the biological roles of *cis*-elements ABRE and recognition sites MYB and MYC in mediating gene transcription under drought condition. TaNF-YB2 protein involves constitution of a heterotrimer with its partners TaNF-YA7 and TaNF-YC7, namely, TaNF-YB2/TaNF-YA7/TaNF-YC7. Overexpression of *TaNF-YB2* confers plants improved drought tolerance through modulating stomata movement, leaf water retention capacity, osmolytes accumulation, and ROS homeostasis. *TaNF-YB2* elicits expression of the P5CS gene *P5CS2* and the AE genes *TaSOD1*, *TaCAT5*, and *TaPOD5*. Knockdown expression of these stress-responsive genes negatively modulated proline accumulation and ROS homeostasis. The transcripts of *TaNF-YB2* and its downstream stress defensive genes are highly correlated with the plant productivity of wheat cultivars under drought condition, with haplotype TaNF-YB2-Hap1 conferring improved gene transcripts and plant drought tolerance. *TaNF-YB2* is an essential modulator in plant drought adaptation and a valuable target for molecular breeding drought-tolerant cultivars in *T. aestivum*.

### Electronic supplementary material

Below is the link to the electronic supplementary material.

Supplementary Material 1

Supplementary Material 2

## Data Availability

The datasets generated and/or analyzed during the current study are available from the corresponding author on reasonable request.

## Abbreviations

NF-Y: Nuclear factor Y type
TF: Transcription factor
Y-2H: Yeast two-hybrid
BiFC: Biomolecular fluoresence complementation
Co-IP: Co-immunoprecipitation
ROS: Reactive oxygen species
AE: Antioxidant enzymes
SOD: Superoxide dismutase
CAT: Catalase
POD: Peroxidase
MDA: Malondialdehyde
P5CS: Pyrroline-5-carboxylate synthase
ABA: Abscisic acid
CBF: CCAAT-binding box
ER: Endoplasmic reticulum
GFP: Green fluorescent protein
YFP: Yellow fluorescent protein
ORF: Open reading frame
WT: Wild type
MS: Murashige and Skoog
PEG-6000: Polyethylene glycol 6000
GUS: β-glucuronidase
X-Gluc: 5-bromo-4-chloro-3-indolyl β- D –glucuronide
EDTA: Ethylenediamine tetraacetic acid
NCBI: National Center for Biotechnology Information
GAPDH: Glyceraldehyde-3-phosphate dehydrogenase
DAPI: 4’,6-Diamidino-2-phenylindole, dihydrochloride
WLR: Leaf water losing rate
SCR: Stomata closing rates
NBT: Nitro blue tetrazolium
DAB: 3.3’-diaminobenzidine
SNP: Single nucleotide polymorphism
