# Supplementary figures and images for: Transcription factor TaNF-YB2 interacts with partners TaNF-YA7/YC7 and transcriptionally activates distinct stress-defensive genes to modulate drought tolerance in T. Aestivum
Source: BMC Plant Biol. 2024 Jul 25;24:705. doi: 10.1186/s12870-024-05420-x (PMC11270858; doi:10.1186/s12870-024-05420-x)

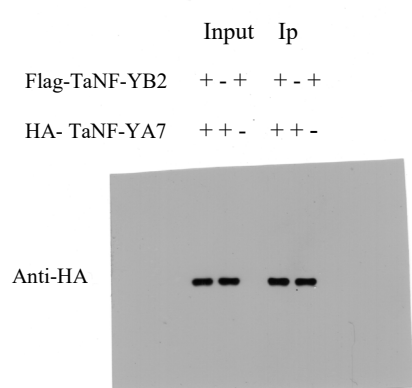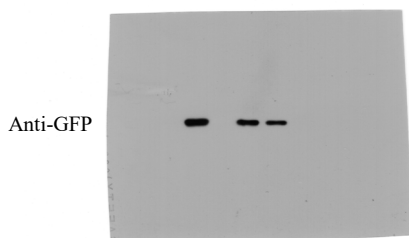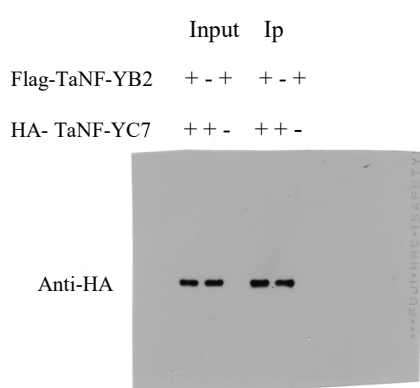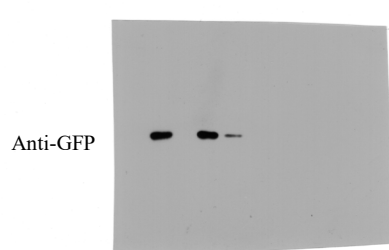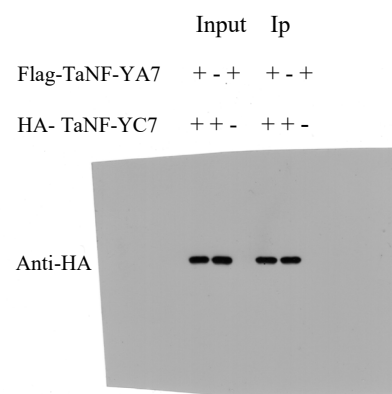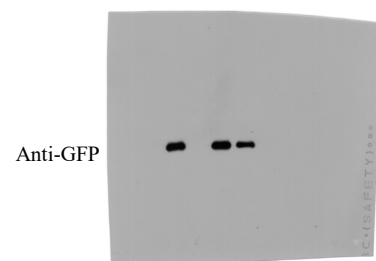

Original, uncropped blots for the Co-IP assay were displayed

Supplement: Supplementary file 1 — Supplementary Material 1 [file 12870_2024_5420_MOESM1_ESM.pdf]
