# Supplementary material for: Transcription factor TaNF-YB2 interacts with partners TaNF-YA7/YC7 and transcriptionally activates distinct stress-defensive genes to modulate drought tolerance in T. Aestivum
Source: BMC Plant Biol. 2024 Jul 25;24:705. doi: 10.1186/s12870-024-05420-x (PMC11270858; doi:10.1186/s12870-024-05420-x)
Supplement: Supplementary file 2 — Supplementary Material 2 [file 12870_2024_5420_MOESM2_ESM.pdf]

Table S1 PCR primers used in this study

Purpose	Accession number	Size of product	Forward primer (5´-)	Reverse primer (5´-)
TaNF-YB2 location cassette	TraesCS2B02G199800	678 bp	tttaagcttatgccggactccgacaac	tttggatccacatgccctgtcttgcct
TaNF-YB2 expression	TraesCS2B02G199800	923 bp	tgaccggagaagggaggagttg	tagcccatttacagtagcgagct
Tatubulin expression	U76558	364 bp	agaacactgttgtaaggctcaac	gagctttactgcctcgaacatgg
TaGAPDH expression	EU022331.1	432bp	ctgccttgctcctcttgctaa	cttgatggaaggaccagcaac
TaNF-YB2 promoter	TraesCS2B02G199800	1798bp	gctgcctagatatggaggcg	ccggagtcgttgtcggagtc
TaNF-YB2-D1-*GUS*	TraesCS2B02G199800	263bp	aaggtacctcgtcaaagtctcaaa	ttccatggcatcgccgcccgccgaag
TaNF-YB2-D2-*GUS*	TraesCS2B02G199800	591bp	ttggtaccataatggaggcaagtgac	ttccatggcatcgccgcccgccgaag
TaNF-YB2-D3-*GUS*	TraesCS2B02G199800	1112bp	ttggtaccgtgtttaaattttagac	ttccatggcatcgccgcccgccgaag
TaNF-YB2-D4-*GUS*	TraesCS2B02G199800	1469bp	ttggtaccaagagaagtaaaactgc	ttccatggcatcgccgcccgccgaag
TaNF-YB2-D5-*GUS*	TraesCS2B02G199800	1771bp	ttggtacctagatatggaggcggg	ttccatggcatcgccgcccgccgaag
TaNF-YB2 bait in two-hyb	tplb0013d06	678 bp	tttggatccatatgccggactccgacaac	tttctgcaggtcatgccctgtcttgcct
TaNF-YA7 bait in two-hyb	AK333451	621 bp	aaagaattcatgacttctgtcgccgac	aaaggatccctcattcatggttccccga
TaNF-YA2 prey in two-hyb	AK333603	630 bp	aaagaattcatgaagcgagctcgtgga	aaaggatcccttacctcatcatggaagc
TaNF-YA3 prey in two-hyb	tplb0037n06	807 bp	aaagaattcatgagtggcatgggatcgc	aaaggatccctcacgcctgacggagatg
TaNF-YA7 prey in two-hyb	AK333451	621 bp	aaagaattcatgacttctgtcgccgac	aaaggatccctcattcatggttccccga
TaNF-YC1 prey in two-hyb	AK331117	723 bp	aaagaattcgagaaccaccagctg	aaaggatccctcactcggagcttggagg
TaNF-YC2 prey in two-hyb	AK333406	772 bp	aaagaattcatggagccatcctcgcag	aaaggatcccctagtttgagggagactgc
TaNF-YC3 prey in two-hyb	AK333583	1468 bp	aaagaattcatggccgggaagaagagc	aaaggatcccctacggctccttcaccac
TaNF-YC4 prey in two-hyb	tplb0006o07	600 bp	aaagaattcatggagcccaaatccacca	aaaggatcccttactgctgcgggacgta
TaNF-YC5 prey in two-hyb	tpl0008i06	1233 bp	aaagaattcatggccgggaagaagcaa	aaaggatcccctatggctccttcgccac
TaNF-YC6 prey in two-hyb	tplb0009e11	555 pb	aaagaattcatggctcccagatcggag	aaaggatcccacccggatacctatggtga
TaNFYC7 prey in two-hyb	KJ862216	768 bp	aaagaatccatggaaccgtcttcacaa	aaacctagggccgggggttgctcacaa
TaNFYC8 prey in two-hyb	KM078730	768 bp	aaaggatccgtatgaggaagaagctgggc	aaactgcagcctaatcctcattgtcgta
TaNF-YB2 prey in one-hyb	tplb0013d06	678 bp	tttgaattcatgccggactccgacaac	tttggtacctcatgccctgtcttgcct
TaP5CS2 bait in one-hyb	KM523670	120 bp	aaacacgtgatggtggactgaacacc	aaaggtaccttttcctcgtcgccgca
TaSOD1 bait in one-hyb	FJ890986	102 bp	aaacacgtgatggttttcttttcgtgcgttc	aaaggtaccatacagtccttgc
TaCAT5 bait in one-hyb	KP892532	153 bp	aaacacgtgatggatcctgcctgaatctata	aaaggtaccgtgggcgtacgtgtgctc
TaPOD5 bait in one-hyb	AJ878510	122 bp	aaacacgtgatggacggagatttagaaggca	aaaggtacctcgcagaaagagagagac
TaNF-YB2-cYFP	TraesCS2B02G199800	678bp	aaatctagaatgccggactccgaca	aaaggatccgcatgccctgtcttgcct
TaNF-YA7-nYFP	AK333451	621bp	aaaggatccatgacttctgtcgccga	aaaggtaccacattcatggttccccg
TaNF-YC7-nYFP	KJ862216	771bp	aaaggatccatggaaccgtcttcacaa	aaaggtaccgccgcttccagattgatg
TaNF-YC7-cYFP	KJ862216	771bp	aaaggatccatggaaccgtcttcacaa	aaaggtaccgccgcttccagattgatg

CaMV35S::TaNF-YB2	TraesCS2B02G199800	1798bp	gctgcctagatatggaggcg	ccggagtcgttgtcggagtc
CaMV35S$_{pro}$::TaNF-YB2 effector	TraesCS2B02G199800	678bp	ccatgccggactccgacaa	ggtcttgccctgtcttgcct
TaNF-YB2 overexpression cassette	TraesCS2B02G199800	730 bp	aaaccatggcggactccgacaacg	aaaggttaccagtaaaacagatac
TaNF-YB2 knockdown cassette	TraesCS2B02G199800	867 bp	tttccatggaataaaattgaccgat	tttggtgaccggagaagggaggagt
TaP5CS1 expression	AB193551	154 bp	gcacgtggacctgtgggtgttg	gttttcgcggaatccttaccacg
TaP5CS2 expression	KM523670	120 bp	gctcttacgagggaaaggg	acttcagagggttttcgcgg
TaP5CS3 expression	KT868850	78 bp	ctcttacgagggaaagggcaa	tcattgcaaaggaaggctc
TaP5CS4 expression	KT218497	79 bp	caagttgataggtatttctgaa	aataaggtatctgttgcctcaa
TaP5CS5 expression	AY888045	132 bp	tggtcactacagatgataaagt	tacttatgccaacctcagcacc
TaSOD1 expression	FJ890986	102 bp	gacgctgatgatcttggcaagg	atcttagccctggagcccgatg
TaSOD2 expression	FJ890987	117 bp	ccccatggactatcaaactcgt	gtcaagtctagctccacttgagt
TaSOD3 expression	JQ613154	126 bp	tgcagttgttgggagagcgt	gtcaggccaacaacaccacat
TaSOD4 expression	AF092524	110 bp	gggcactattccactgtcgg	gcaacgcaggcacagtaacaa
TaSOD5 expression	KR069092	120 bp	gtacggccagcgcacgtgt	gggttaaggttccgtaggtg
TaSOD6 expression	KC158224	141bp	caggaccctcttgtgaccaaa	atatttccagttcaccaccttcc
TaCAT1 expression	D86327	159 bp	gcgagaagatggtgatcga	aggagagccagatggccttg
TaCAT2 expression	X94352	150 bp	gcctcagctggcgtcgatc	acgcgctgacgacaccccac
TaCAT3 expression	GU984379	189 bp	caaggccatctggctctcct	tgtcagcctgcacgatccc
TaCAT4 expression	HQ860268	170 bp	ggagaagacgaggatcaagaag	acttggagaggaagtcgatc
TaCAT5 expression	KP892532	153 bp	ccagtggctcacccgcctcggt	acaccaactatcattgttcatc
TaCAT6 expression	KP892533	154 bp	gggcagaagctggcgtcgcgg	ttcatggctacacccacagag
TaPOD1 expression	AF387866	124 bp	gcacacagaatgcaaaggca a	cgtgaagtacctacttcatgcc
TaPOD2 expression	AB518867	165 bp	cacgaagccagcgggacttc	tctgcacaaaggacggccac
TaPOD3 expression	XM_044596808	119 bp	ggctcagctgctccaaggt	tggtatgataatcggtactgg
TaPOD4 expression	XM_044466229	132 bp	tacgagcagcagcaaatcat	gccctatgcatctggacagag
TaPOD5 expression	AJ878510	122 bp	acaggcgtgtccaggatatc	aacacaccggcttaacggtc
TaPOD6 expression	MW030685	149 bp	cttcaagaccttcgacaccg a	cggcgaagaactcgtccttgaa
TaPOD7 expression	MT166266	141 bp	ggaactcgtcaagctctacg	ttgtggttaaccctcctgcag
TaPOD8 expression	X56011	111 bp	agatcaggctcagctgctcc	attggccactggttctgtgg
TaPOD9 expression	X53675	204 bp	ttgtacacggagtgaagatgtac	ccctgcacgtctaggattatc
TaP5CS2 knockdown cassette	KM523670	1128 bp	aaaccatggtctggtgagactctc	aaaggtaaccaggaaagatagcaa
TaSOD1 knockdown cassette	FJ890986	456 bp	aaaccatggcacagccttcaccat	aaaggtcaccttagccctggagccc
TaCAT5 knockdown cassette	KP892532	792 bp	aaaccatggatgcctgtgtttttcatc	aaaggtgacctcatgcaagcagaagg
TaPOD5 knockdown cassette	AJ878510	1023 bp	aaaccatggatggcgtccagagcagca	aaaggtaacctcacatgtcggcggcgatc
CaMV35S$_{pro}$::TaNF-YB2 effector	TraesCS2B02G199800	678 bp	aaaggatccccatgccggactccgacaa	aaactgcagggtcttgccctgtcttgcct

CaMV35S$_{pro}$*::TaNF-YA7 effector*	AK333451	621bp	aaaaagcttcatgacttctgtcgccga	aaactgcagggtctttcatggttccccgact
CaMV35Spro::TaNF-YC7 effector	KJ862216	771bp	aaaaagcttatggaaccgtcttcacaa	aaagaattctctgccgcttccagattg
CaMV35S$_{pro}$*::LUC*	DI336552	63bp	gctctagataacttcgtata	atgtcgaggctcagcaggaa
TaP5CS2$_{pro}$*::LUC*	KM523670	1903bp	tggactgaacacccccccc	tttttcctcgtcgccgca
TaSOD1$_{pro}$*::LUC*	FJ890986	1825bp	ttttcttttcgtgcgttc	atacagtccttgcttcgt
TaCAT5$_{pro}$*::LUC*	KP892532	1761bp	atcctgcctgaatctata	gtgggcgtacgtgtgctc
TaPOD5$_{pro}$*::LUC*	AJ878510	1765bp	acggagatttagaaggca	tcgcagaaagagagagac

Table S2 Information of the forty-five wheat cultivars examined for a field experiment

Cultivar name	Registration authorization and number	Registration date	Breeding organization
BN 207	National approval wheat cultivar, 20200106	2020	Hebei Bo Genetic Technology Co., Ltd.
HN 085	HeBei approval wheat cultivar, 20190014	2019	Hebei Agricultural University
H 4399	HeBei approval wheat cultivar, 2008002	2008	Institute of Dryland Agriculture, Hebei Academy of Agriculture and Forestry Sciences
XM 13	National approval wheat cultivar, 2016021	2016	Xingtai Academy of Agricultural Sciences
JM 418	National approval wheat cultivar, 2016026	2016	Institute of Grain and Oil Crops, Hebei Academy of Agricultural Sciences
JM 691	National approval wheat cultivar, 20230157	2023	Institute of Grain and Oil Crops, Hebei Academy of Agricultural Sciences
SM 26	National approval wheat cultivar, 20180052	2018	Shijiazhuang Academy of Agricultural and Forestry Sciences, Hebei Wheat Engineering Technology Research Center
HM 26	National approval wheat cultivar, 20230153	2023	Handan Academy of Agricultural Sciences
ML-1	HeBei approval wheat cultivar, 20218011	2021	Hebei Dadi Seed Industry Co., Ltd., Shijiazhuang Academy of Agricultural and Forestry Sciences, Xinji Malan Farm
HN 8324	HeBei approval wheat cultivar, 20230006	2023	Hebei Agricultural University
JM 585	HeBei approval wheat cultivar, 2013004	2013	Institute of Grain and Oil Crops, Hebei Academy of Agricultural Sciences
S 4366	HeBei approval wheat cultivar, 20198019	2019	Shijiazhuang Academy of Agricultural and Forestry Sciences, Shijiazhuang Wanfeng Seed Industry Co., Ltd.
HN 6133	HeBei approval wheat cultivar, 20210020	2021	Hebei Agricultural University
SN 086	HeBei approval wheat cultivar, 20198017	2019	Hebei Dadi Seed Production Co., Ltd.
SM 32	National approval wheat cultivar, 20220092	2022	Shijiazhuang Academy of Agriculture and Forestry Sciences
SM 28	National approval wheat cultivar, 20180063	2018	Shijiazhuang Academy of Agricultural and Forestry Sciences, Hebei Wheat Engineering Technology Research Center
HG 35	Tianjin approval wheat cultivar, 2007003	2019	Institute of Dryland Agriculture, Hebei Academy of Agriculture and Forestry Sciences
SM 22	HeBei approval wheat cultivar, 2013002	2013	Shijiazhuang Academy of Agricultural and Forestry Sciences, Hebei Wheat Engineering Technology Research Center
JM 325	National approval wheat cultivar, 20210063	2021	Institute of Grain and Oil Crops, Hebei Academy of Agricultural Sciences
HM 19	National approval wheat cultivar, 20180049	2018	Handan Academy of Agricultural Sciences
DD 1912	National approval wheat cultivar, 20220085	2022	Hebei Dadi Seed Production Co., Ltd.
KN 1002	HeBei approval wheat cultivar, 20210012	2021	Genetics and Development, Chinese Academy of Sciences
HN 7008	HeBei approval wheat cultivar, 20210033	2021	Hebei Agricultural University
H 6172	National approval wheat cultivar, 2003036	2003	Handan Academy of Agricultural Sciences
J 5265	National approval wheat cultivar, 2009016	2009	Institute of Grain and Oil Crops, Hebei Academy of Agricultural Sciences

H 444	HeBei approval wheat cultivar, 2012002	2012	Institute of Dryland Agriculture, Hebei Academy of Agriculture and Forestry Sciences
ND 399	HeBei approval wheat cultivar, 2012004	2012	College of Agronomy and Biotechnology, China Agricultural University, Hebei Jincheng Seed Industry Co., Ltd.
SM 25	HeBei approval wheat cultivar, 2016013	2016	Shijiazhuang Academy of Agricultural and Forestry Sciences, Hebei Wheat Engineering Technology Research Center
S 4185	National approval wheat cultivar, 990007	1999	Shijiazhuang Academy of Agricultural and Forestry Sciences
HN 9204	National approval wheat cultivar, 20220051	2022	Hebei Agricultural University
ZM 155	HeBei approval wheat cultivar, 2012005	2012	Institute of Crop Sciences, Chinese Academy of Agricultural Sciences
HN 6426	HeBei approval wheat cultivar, 20190018	2019	Hebei Agricultural University
SN 956	HeBei approval wheat cultivar, 2016016	2016	Shijiazhuang Dadi Seed Industry Co., Ltd., Shijiazhuang New Wheat Variety New Technology Research Institute
JM 22	National approval wheat cultivar, 2006018	2006	Crop Research Institute of Shandong Academy of Agricultural Sciences
GY 2018	HeBei approval wheat cultivar, 2008007	2008	Gaocheng Institute of Agricultural Sciences
GY 5818	HeBei approval wheat cultivar, 20190010	2019	Gaocheng District Institute of Agricultural Sciences, Shijiazhuang City
HN 5102	HeBei approval wheat cultivar, 20190022	2019	Hebei Agricultural University
JM 120	HeBei approval wheat cultivar, 2016019	2016	Institute of Grain and Oil Crops, Hebei Academy of Agricultural Sciences
GY 5766	HeBei approval wheat cultivar, 2014002	2014	Gaocheng Institute of Agricultural Sciences
SL 02-1	National approval wheat cultivar, 2007016	2007	Hebei Normal University, Luancheng County Seed Farm
GY 5218	HeBei approval wheat cultivar, 2015005	2015	Gaocheng District Institute of Agricultural Sciences, Shijiazhuang City
H 6172	National approval wheat cultivar, 2003036	2003	Handan Academy of Agricultural Sciences, Hebei Province
SY 20	National approval wheat cultivar, 2011011	2011	Shijiazhuang Academy of Agriculture and Forestry Sciences
ZXM 99	National approval wheat cultivar, 20180053	2018	Hebei Zhongxin Seed Technology Co., Ltd.
SN 952	HeBei approval wheat cultivar, 20208017	2020	Hebei Dadi Seed Production Co., Ltd.

Table S3 *cis*-acting regulatory elements situated in the *TaNF-YB2* promoter

Element	Distance from ATG (±strand)	Motif sequence	Function
A-box	384-<br>286+	CCGTCC<br>CCGTCC	*cis*-acting regulatory element
TATA-box	975-<br>815-<br>815-<br>815-	TATA<br>TATATAA<br>TATATA<br>TATA	core promoter element around −30 of transcription start
CAAT-box	849-<br>690-<br>623-<br>145-	CCAAT<br>CAAAT<br>CCAAT<br>CAAT	common *cis*-acting element in promoter and enhancer regions
ABRE	1395-<br>473+<br>507-<br>333-<br>1002+<br>330+<br>487-<br>323-<br>1351+<br>1004+	GACACGTGGC<br>ACGTG<br>ACGTG<br>ACGTG<br>GCAACGTGTC<br>CGCACGTGTC<br>ACGTG<br>ACGTG<br>ACGTG<br>ACGTG	ABA, drought responsive element
ABRE3a	486-	TACGTG	ABA, drought responsive element
ABRE4	486+	CACGTA	ABA, drought responsive element
MYB	657-<br>88-	CAACCA<br>CAACCA	ABA ,drought responsive element
MYB recognition site	544-	CCGTTG	drought responsive element
MYC	1739+<br>763-	CATTTG<br>CATTTG	drought and salt stress responsive element
AAGAA-motif	1036-	GAAAGAA	*cis*-acting element involved in the abscisic acid responsiveness
CCGTCC-box	384-<br>286+	CCGTCC<br>CCGTCC	*cis*-acting regulatory element related to meristem expression
CAT-box	1399+<br>663+<br>1289-<br>213+	GCCACT<br>GCCACT<br>GCCACT<br>GCCACT	*cis*-acting regulatory element related to meristem expression
CCAAT-box	544+	CAACGG	MYBHv1 binding site
CGTCA-motif	1215-<br>142-<br>321+<br>505+<br>252+	CGTCA<br>CGTCA<br>CGTCA<br>CGTCA<br>CGTCA	*cis*-acting regulatory element involved in the MeJA-responsiveness
TGACG-motif	1215+<br>142+<br>321-<br>505-<br>252-	TGACG<br>TGACG<br>TGACG<br>TGACG<br>TGACG	*cis*-acting regulatory element involved in the MeJA-responsiveness

Table S4 Drought-related *cis*-acting elements situated in *TaP5CS2*, *TaSOD1*, *TaCAT5* and *TaPOD5* promoters

Gene Name	Site Name	Sequence	Position	Strand	Organism	Function
TaP5CS2	CAAT-box	CAAT	274	4 (-)	*Nicotiana glutinosa*	common cis-acting element in promoter and enhancer regions
		CAAT	385	4 (-)	*Nicotiana glutinosa*	
		CAAT	467	4 (+)	*Nicotiana glutinosa*	
		CAAT	664	4 (-)	*Nicotiana glutinosa*	
		CAAT	776	4 (+)	*Nicotiana glutinosa*	
		CAAAT	1058	5 (-)	*Pisum sativum*	
		CAAT	1050	4 (+)	*Nicotiana glutinosa*	
		CAAT	1167	4 (-)	*Nicotiana glutinosa*	
		CAAT	1191	4 (-)	*Nicotiana glutinosa*	
		CAAT	1200	4 (+)	*Nicotiana glutinosa*	
		CAAT	1327	4 (+)	*Nicotiana glutinosa*	
		CAAAT	1408	5 (+)	*Pisum sativum*	
	TATA-box	TATA	261	4 (+)	*Arabidopsis thaliana*	core promoter element around -30 of transcription start
		TATA	1392	4 (-)		
		TATA	1573	4 (-)		
	ABRE	ACGTG	516	5 (+)	*Arabidopsis thaliana*	cis-acting element involved in the abscisic acid responsiveness
		TACGGTC	797	7 (-)		
		ACGTG	839	5 (-)		
		TACGGTC	1348	7 (-)		
		ACGTG	1644	5 (+)		
	DRE core	GCCGAC	1292	6 (+)	*Arabidopsis thaliana*	
		GCCGAC	1331	6 (-)		
	MBS	CAACTG	280	6 (-)	*Arabidopsis thaliana*	MYB binding site involved in drought-inducibility
		CAACTG	562	6 (-)		
		CAACTG	943	6 (-)		
	MYB	CAACCA	618	6 (+)	*Arabidopsis thaliana*	ABA ,drought responsive element
	MYB recognition site	CCGTTG	1749	6 (-)	Arabidopsis thaliana	drought responsive element
	MYC	TCTCTTA	1922	7 (-)	Arabidopsis thaliana	drought and salt stress responsive element
TaSOD1	CAAT-box	CCAAT	250	5 (+)	Arabidopsis thaliana	common cis-acting element in promoter and enhancer regions
		CAAT	251	4 (+)	Nicotiana glutinosa	
		CAAT	301	4 (-)	Nicotiana glutinosa	
		CAAAT	569	5 (-)	Pisum sativum	
		CAAT	596	4 (+)	Nicotiana glutinosa	
		CAAT	617	4 (-)	Nicotiana glutinosa	
		CAAT	635	4 (+)	Nicotiana glutinosa	
		CAAAT	658	5 (+)	Pisum sativum	
		CAAAT	706	5 (+)	Pisum sativum	
		CAAT	720	4 (-)	Nicotiana glutinosa	
		CAAAT	736	5 (+)	Pisum sativum	
		CAAAT	1390	5 (-)	Pisum sativum	
		CAAT	1451	4 (+)	Nicotiana glutinosa	
		CAAT	1555	4 (-)	Nicotiana glutinosa	
		CAAAT	1618	5 (-)	Pisum sativum	

Gene Name	Site Name	Sequence	Position	Strand	Organism	Function
TaSOD1	**CAAT-box**	CAAAT	1625	5 (-)	Pisum sativum	common cis-acting element in promoter and enhancer regions
		CAAAT	1642	5 (-)	Pisum sativum	
		CAAAT	1791	5 (-)	Pisum sativum	
		CAAT	1844	4 (-)	Nicotiana glutinosa	
		CAAAT	1929	5 (+)	Pisum sativum	
		CAAT	1934	4 (+)	Nicotiana glutinosa	
		CAAT	1981	4 (-)	Nicotiana glutinosa	
	**DRE core**	GCCGAC	802	6 (-)	Arabidopsis thaliana	
		GCCGAC	1324	6 (+)		
	**MYB**	CAACCA	301	6 (+)	Arabidopsis thaliana	ABA ,drought responsive element
		CAACCA	456	6 (+)		
		CAACCA	618	6 (+)		
		TAACCA	702	6 (+)		
		CAACAG	1534	6 (-)		
		TAACCA	1800	6 (+)		
	**MYC**	CATTTG	568	6 (+)	Arabidopsis thaliana	drought and salt stress responsive element
		CATTTG	706	6 (-)		
	**MYB-binding site**	CAACAG	1534	6 (-)	Nicotiana tabacum	drought responsive element
TaCAT5	**CAAT-box**	CAAAT	181	5 (-)	Pisum sativum	common cis-acting element in promoter and enhancer regions
		CAAAT	299	5 (+)	Pisum sativum	
		CAAAT	325	5 (-)	Pisum sativum	
		CAAT	342	4 (+)	Nicotiana glutinosa	
		CCAAT	349	5 (-)	Arabidopsis thaliana	
		CAAT	858	4 (-)	Nicotiana glutinosa	
		CCAAT	1028	5 (+)	Arabidopsis thaliana	
		CAAT	1029	4 (+)	Nicotiana glutinosa	
		CAAT	1049	4 (+)	Nicotiana glutinosa	
		CAAT	1083	4 (-)	Nicotiana glutinosa	
		CAAAT	1157	5 (+)	Pisum sativum	
		CCAAT	1315	5 (-)	Arabidopsis thaliana	
		CAAAT	1349	5 (+)	Pisum sativum	
	**TATA-box**	TATAAAA	153	7 (-)	Pisum sativum	core promoter element around -30 of transcription start
		TATAA	155	5 (-)	Arabidopsis thaliana	
		TATA	156	4 (+)	Arabidopsis thaliana	
		TATACA	629	6 (-)	Helianthus annuus	
		TATA	631	4 (+)	Arabidopsis thaliana	
		TATA	763	4 (+)	Arabidopsis thaliana	
		TATA	1043	4 (-)	Arabidopsis thaliana	
		TATATA	1373	6 (-)	Arabidopsis thaliana	
		ATATAT	1374	6 (-)	Brassica napus	
		TATA	1375	4 (-)	Arabidopsis thaliana	
	**DRE core**	GCCGAC	1057	6 (+)	Arabidopsis thaliana	
		GCCGAC	1579	6 (+)		
	**DRE1**	ACCGAGA	652	7 (-)	Zea mays	
	**MYB**	TAACCA	840	6 (+)	Arabidopsis thaliana	ABA ,drought responsive element
		TAACCA	1211	6 (+)		
		CAACCA	1601	6 (+)	Arabidopsis thaliana	

Gene Name	Site Name	Sequence	Position	Strand	Organism	Function
		CAACCA	1626	6 (+)		
		TAACCA	1706	6 (+)		
		CAACCA	1745	6 (+)		
	**MYB recognition site**	CCGTTG	1131	6 (+)	Arabidopsis thaliana	drought responsive element
	**ABRE**	ACGTG	492	5 (+)	Arabidopsis thaliana	cis-acting element involved in the abscisic acid responsiveness
		TACGGTC	521	7 (-)		
		ACGTG	839	5 (-)		
	**MYC**	CATTTG	180	6 (+)	Arabidopsis thaliana	drought and salt stress responsive element
		CATGTG	299	6 (-)		
		CATGTG	618	6 (+)		
		CATTTG	1157	6 (-)		
		CATTTG	1244	6 (+)		
TaPOD5	**CAAT-box**	CAAAT	1064	5 (-)	Pisum sativum	common cis-acting element in promoter and enhancer regions
		CAAAT	1110	5 (+)	Pisum sativum	
		CAAT	1410	4 (+)	Nicotiana glutinosa	
		CAAAT	1506	5 (+)	Pisum sativum	
		CAAAT	1514	5 (+)	Pisum sativum	
		CAAT	1535	4 (+)	Nicotiana glutinosa	
		CAAT	1547	4 (+)	Nicotiana glutinosa	
		CAAAT	1760	5 (-)	Pisum sativum	
		CAAT	1765	4 (+)	Nicotiana glutinosa	
	**TATA-box**	TATAA	1495	5 (-)	Arabidopsis thaliana	core promoter element around -30 of transcription start
		TATA	1496	4 (-)	Arabidopsis thaliana	
		ATATAT	1572	6 (-)	Brassica napus	
		TATATA	1573	6 (-)	Arabidopsis thaliana	
		TATA	1575	4 (-)	Arabidopsis thaliana	
		TATA	1580	4 (-)	Arabidopsis thaliana	
		TATA	1588	4 (-)	Arabidopsis thaliana	
		TATACA	1887	6 (-)	Helianthus annuus	
		TATA	1889	4 (-)	Arabidopsis thaliana	
	**MBS**	CAACTG	1466	6 (+)	Arabidopsis thaliana	MYB binding site involved in drought-inducibility
		CAACTG	1702	6 (-)	Arabidopsis thaliana	
	**MYB**	TAACCA	731	6 (+)	Arabidopsis thaliana	ABA ,drought responsive element
		TAACCA	1731	6 (+)		
	**MYB recognition site**	CCGTTG	540	6 (+)	Arabidopsis thaliana	drought responsive element
	**ABRE**	TACGGTC	516	7 (-)	Arabidopsis thaliana	cis-acting element involved in the abscisic acid responsiveness
		ACGTG	517	5 (+)		
		ACGTG	589	5 (+)		
		ACGTG	816	5 (+)		
		TACGGTC	817	7 (-)		
		ACGTG	923	5 (-)		

```
1      ATGCCGGACTCCGACAACGACTCCGGCGGGCCGAGCAACGCGGACTTCTCGTCGCCCAAG
1       M  P  D  S  D  N  D  S  G  G  P  S  N  A  D  F  S  S  P  K

61     GAGCAGGACAGGTTCCTGCCGATCGCCAACGTGAGCCGGATCATGAAGAAGGCGCTGCCG
21      E  Q  D  R  F  L  P  I  A  N  V  S  R  I  M  K  K  A  L  P

121    GCCAACGCCAAGATCTCCAAGGACGCCAAGGAGACGGTGCAGGAGTGCGTGTCCGAGTTC
41      A  N  A  K  I  S  K  D  A  K  E  T  V  Q  E  C  V  S  E  F

181    ATCTCCTTCATCACCGGCGAGGCCTCCGACAAGTGCCAGCGCGAGAAGCGCAAGACCATC
61      I  S  F  I  T  G  E  A  S  D  K  C  Q  R  E  K  R  K  T  I

241    AACGGCGACGACCTGCTCTGGGCCATGACCACGCTCGGCTTCGAGGACTACATGGAGCCG
81      N  G  D  D  L  L  W  A  M  T  T  L  G  F  E  D  Y  M  E  P

301    CTCAGGCTCTACCTCCACAAGTTCCGCGAGCTCGAGGGCGAGAAGGCCGTCGGCGCCGGC
101     L  R  L  Y  L  H  K  F  R  E  L  E  G  E  K  A  V  G  A  G

361    GGCGTTGGCGCGTTACCCTCCCCCGNCGGGTCAGGCTCGCAGCAGAGGGAGTCGACGCCA
121     G  V  G  A  L  P  S  P  X  G  S  G  S  Q  Q  R  E  S  T  P

421    AGGAACAATGGCGGAGGCGGTGAGGCCGGCGGCTACGGCGGCATGTACGGTGGCGCCGGG
141     R  N  N  G  G  G  G  E  A  G  G  Y  G  G  M  Y  G  G  A  G

481    GCAGGAGGAGGCGGCGGCGGCATGTTCATGATGATGGGGCAGCCCATGTACGGGTCCCCG
161     A  G  G  G  G  G  G  M  F  M  M  M  G  Q  P  M  Y  G  S  P

541    CCGGCGGCAGGGTACCAACATCCGCAGCATCACCACCAGATGATGACGGGCGGGCAAGGC
181     P  A  A  G  Y  Q  H  P  Q  H  H  H  Q  M  M  T  G  G  Q  G

601    GGGTACGGCTACGGCGACGCCGGCGCCGGCGGCGGGTCTTCGTCGTCCTCAGGGTTCGGC
201     G  Y  G  Y  G  D  A  G  A  G  G  G  S  S  S  S  S  G  F  G

661    AGGCAAGACAGGGCATGA
221     R  Q  D  R  A  *
```

Fig. S1 The open reading frame of *TaNF-YB2* and its corresponding translated amino acids.

The translation start codon ATG and termination codon TAG of *TaNF-YB2* are highlighted using boxes. The functional domain specified by the NF-YB proteins in plant species is underlined.

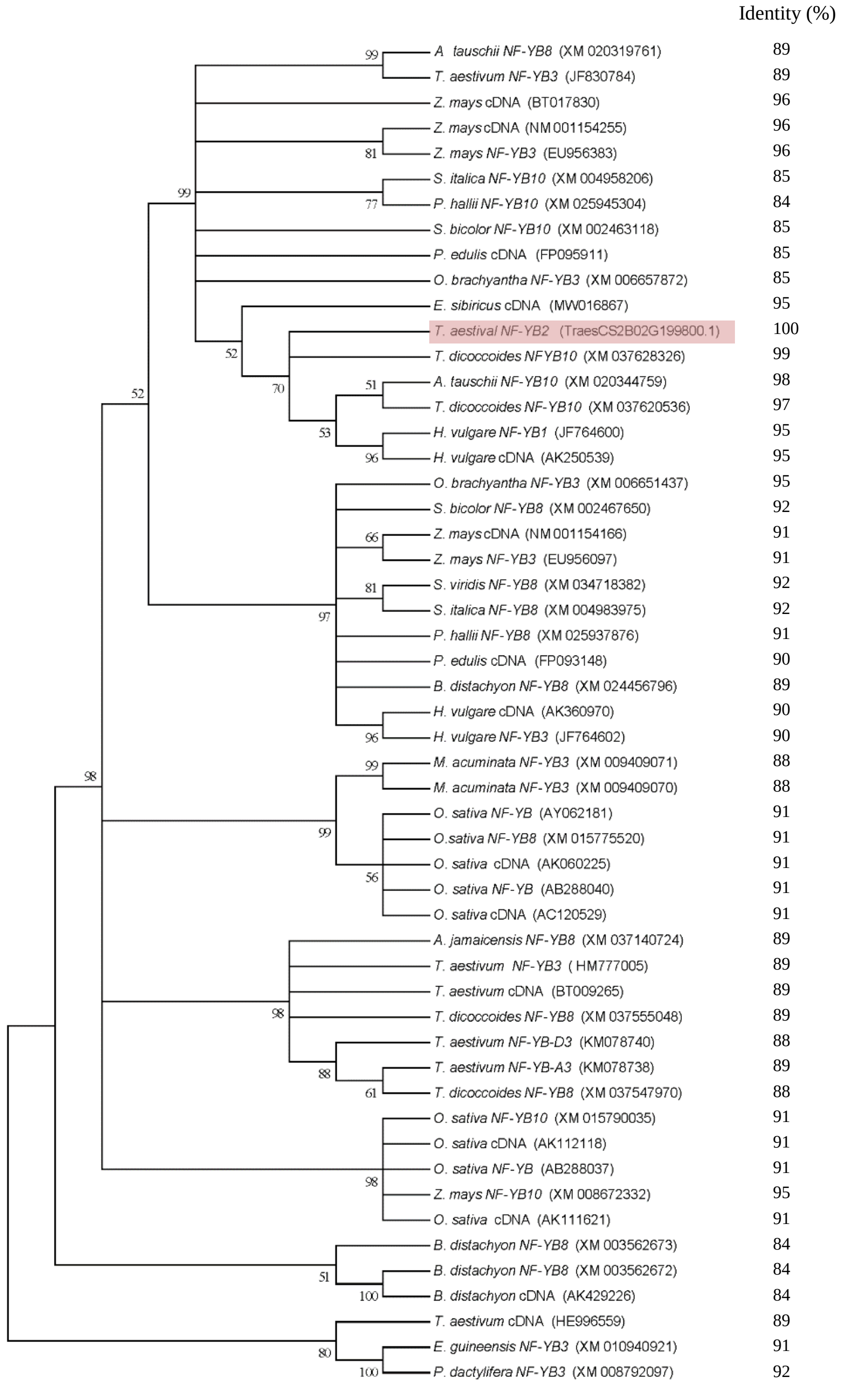

Fig. S2 Phylogenetic relationships among *TaNF-YB2* and its homologous genes across various plant species.

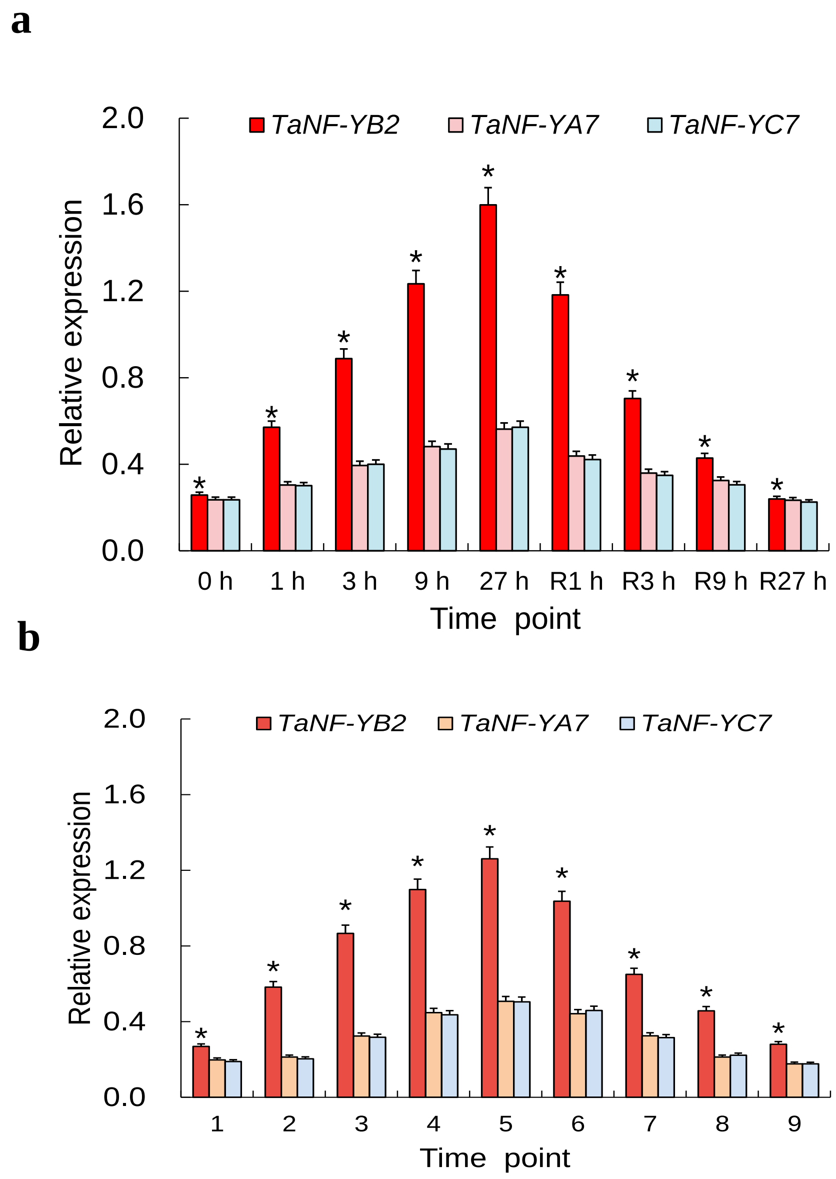

Fig. S3 The expression of *TaNF-YB2*, *TaNF-YA7*, and *TaNF-YC7* under drought stress conditions.

(a) Expression patterns of *TaNF-YB2*, *TaNF-YA7*, and *TaNF-YC7* in roots. (b) Expression patterns of *TaNF-YB2*, *TaNF-YA7*, and *TaNF-YC7* in leaves.

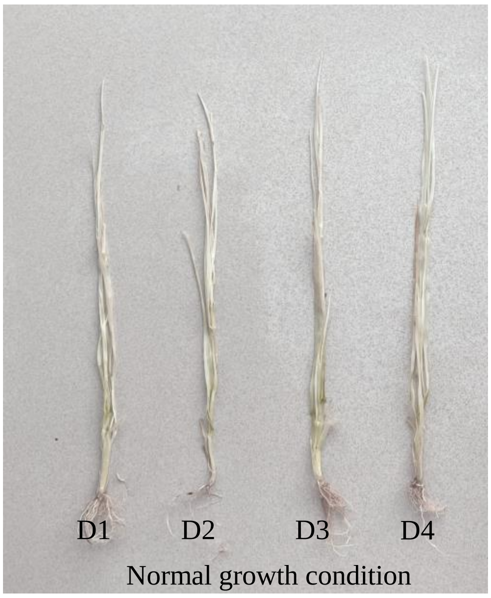

Fig. S4 GUS histochemical staining results in which reporter gene was driven by different length of *TaNF-YB2* promoters under normal growth condition

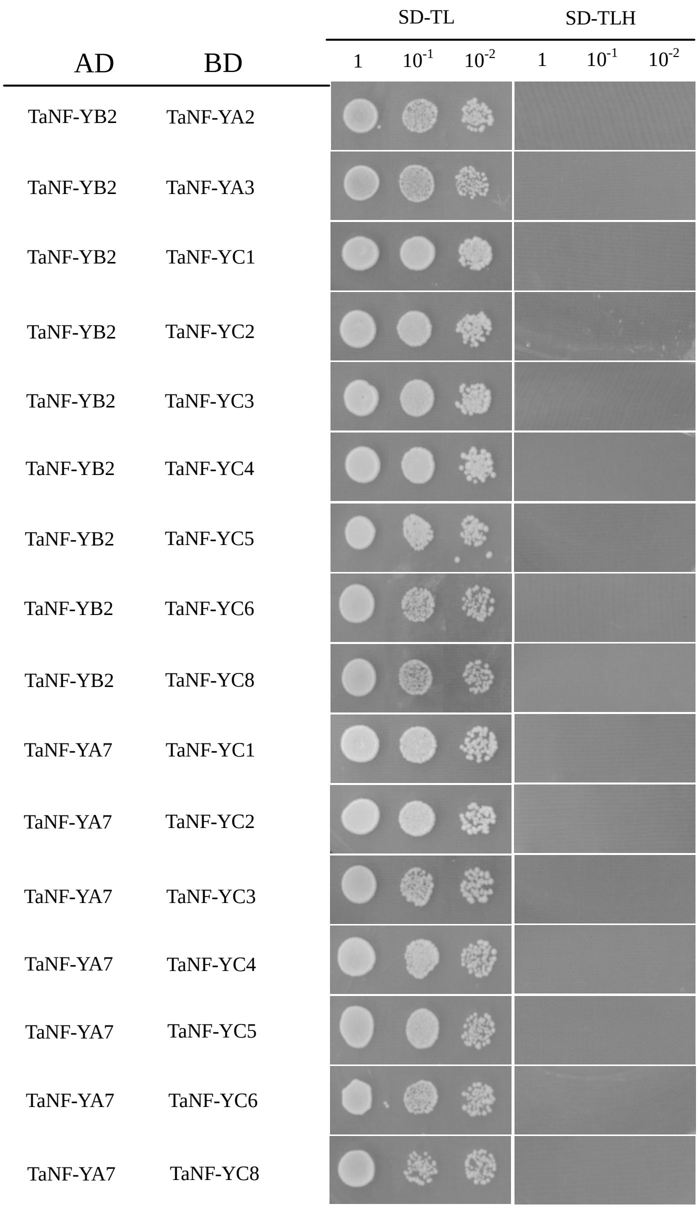

Fig. S5 Yeast two-hybrid assay results between the TaNF-YB2 protein and the members in subfamilies of NF-YA and NF-YC in *T. aestivum*.

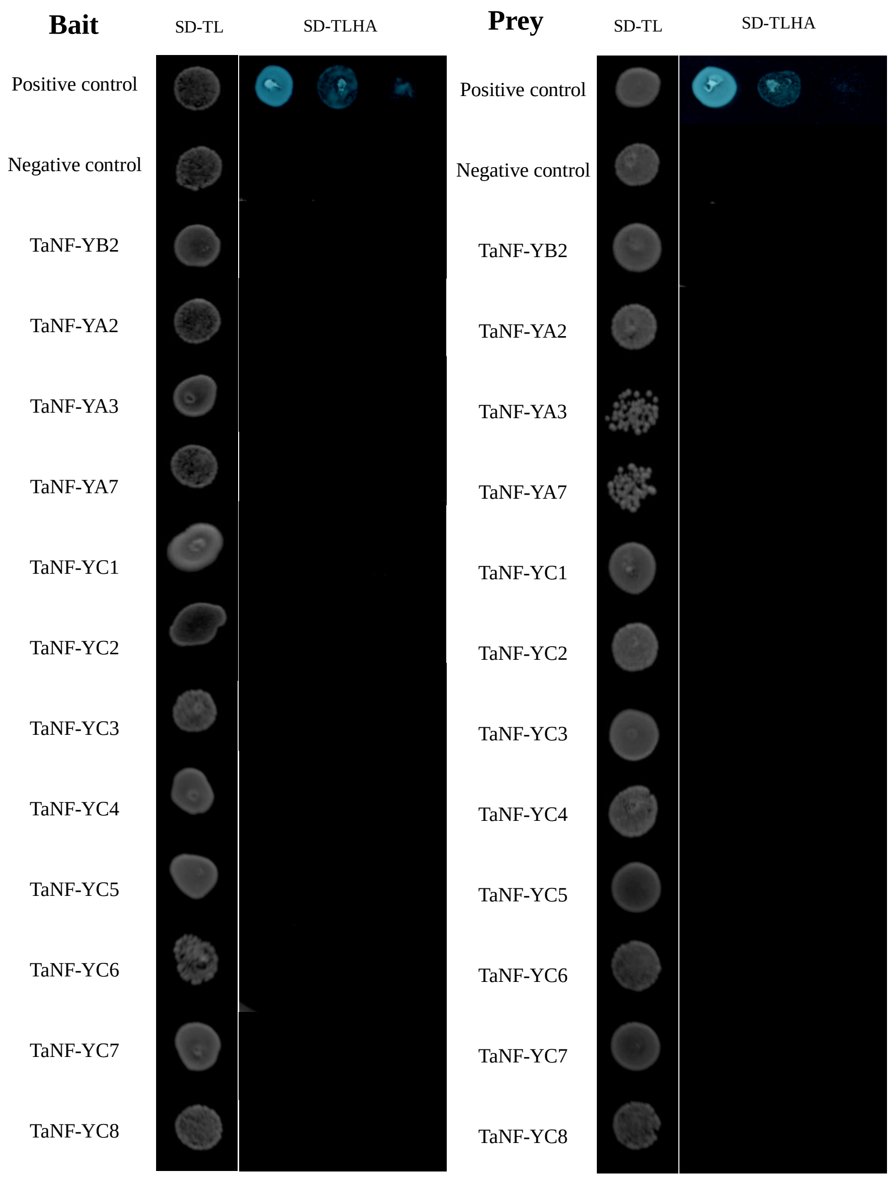

Fig. S6 Auto-activation detection of NF-Y candidate proteins in yeast cells.

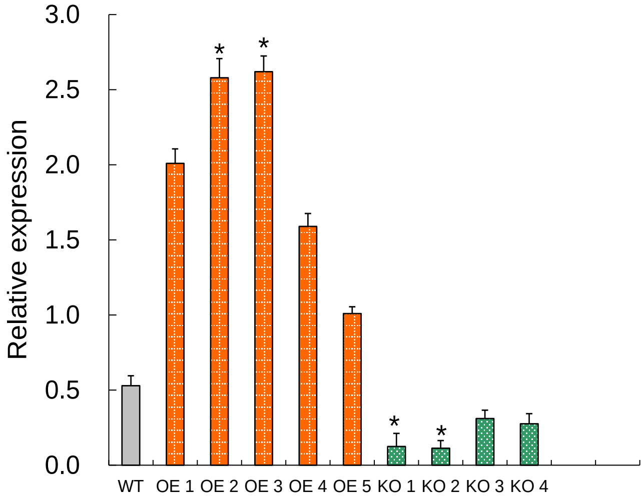

Fig. S7 Expression levels of the target genes in transgenic wheat lines of *TaNF-YB2*.

Expression levels of target gene in *TaNF-YB2* transgenic lines. Data shown are means ± standard deviation (n = 3). The expression values of target genes were normalized by *Tatubulin* and *TaGAPDH*, two constitutive genes in *T. aestivum* species, whose expression levels were set as 1.

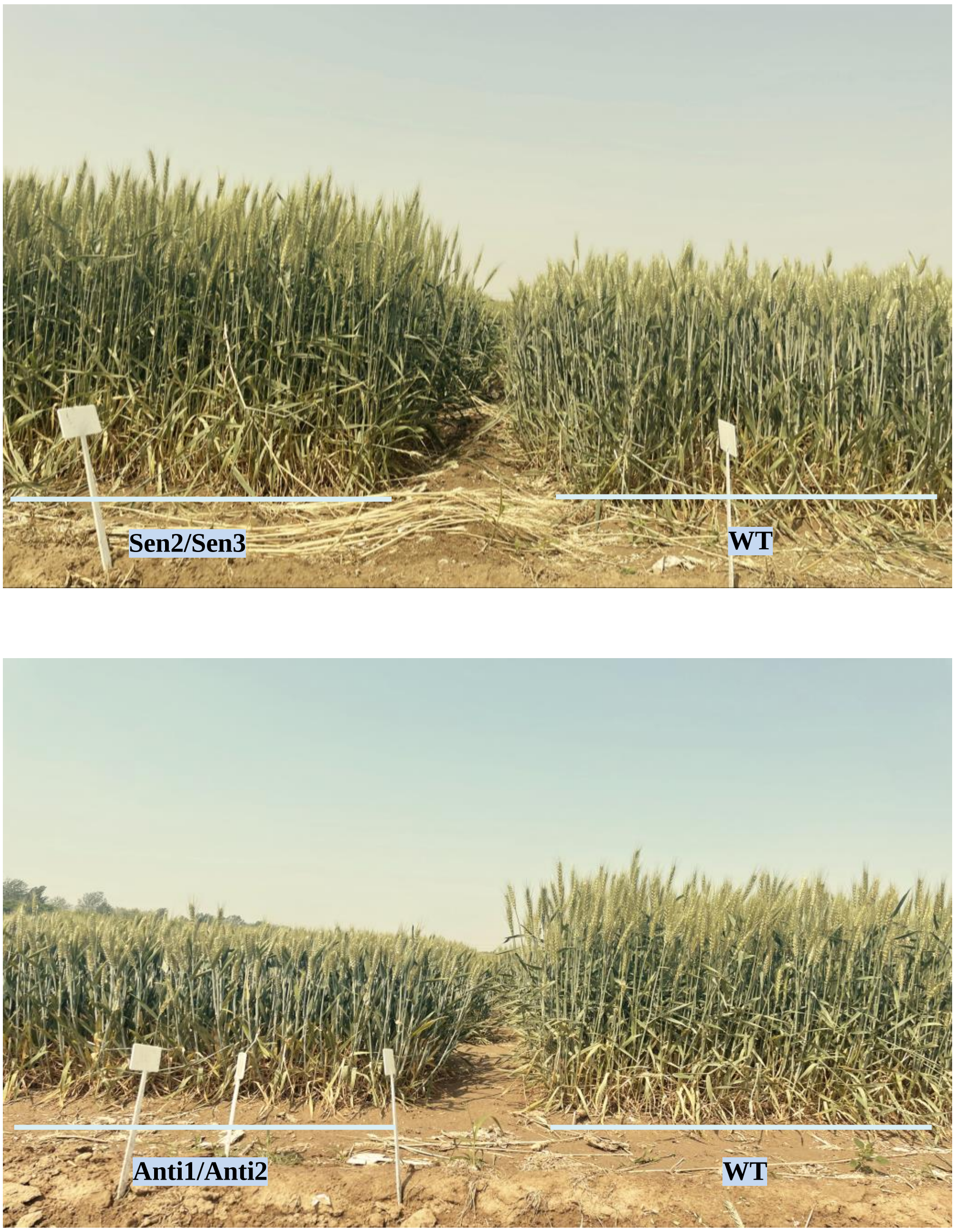

Fig. S8 Phenotypes of TaNF-YB2 transgenic lines at seed filling stage.

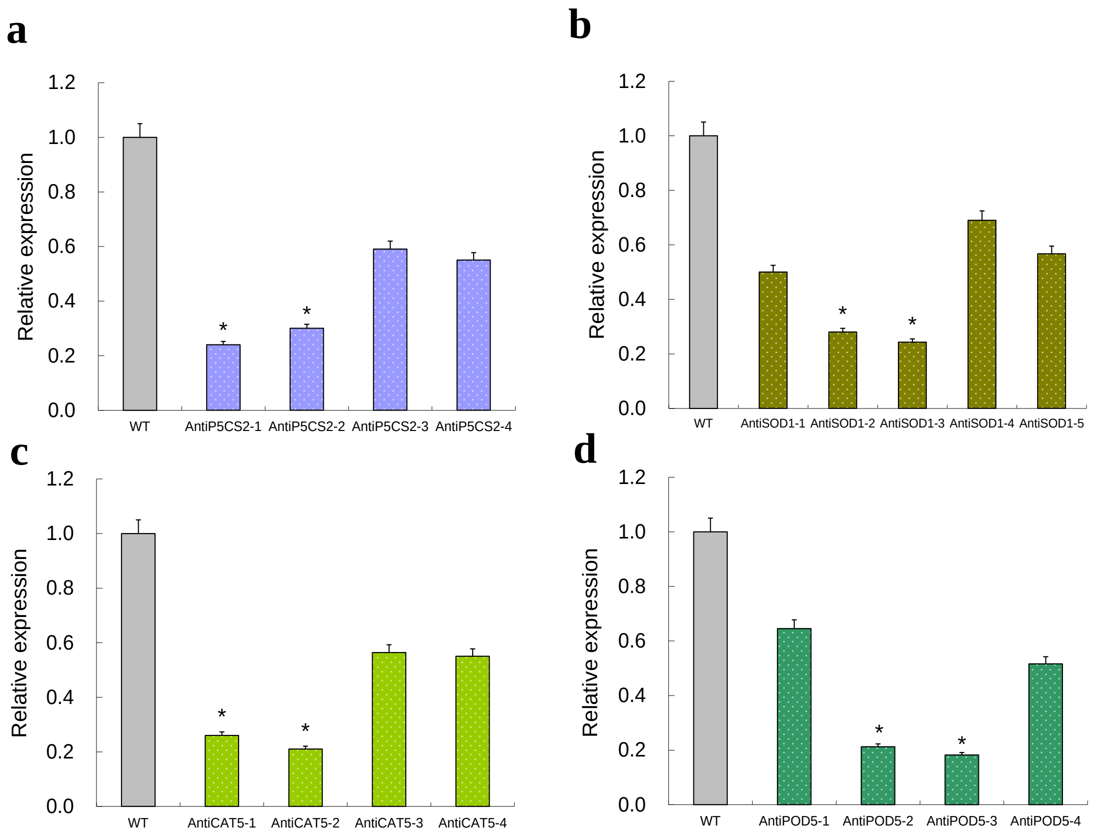

Fig. S9 Expression levels of the target genes in transgenic lines with knockdown expression of distinct osmotic stress responsive-associated genes.

(a) Expression levels of target gene for *TaP5CS2* transgenic lines. (b) Expression levels of target gene for *TaSOD1* transgenic lines. (c) Expression levels of target gene for *TaCAT5* transgenic lines (d) Expression levels of target gene for *TaPOD5* transgenic lines. In (a) AntiP5CS2-1 to AntiP5CS2-4, four lines with *TaP5CS2* knockdown expression. In (b) AntiSOD1-1 to AntiSOD1-5, five lines with *TaSOD1* knockdown expression. In (c) AntiCAT5-1 to AntiCAT5-4, four lines with *TaCAT5* knockdown expression. In (d) AntiPOD5-1 to AntiPOD5-4, four lines with *TaPOD5* knockdown expression. In (a)-(d) WT, wild type. Data shown are means ± standard deviation (n = 3). The expression values of target genes were normalized by *Tatubulin* and *TaGAPDH*, two constitutive genes in *T. aestivum* species, whose expression levels were set as 1.

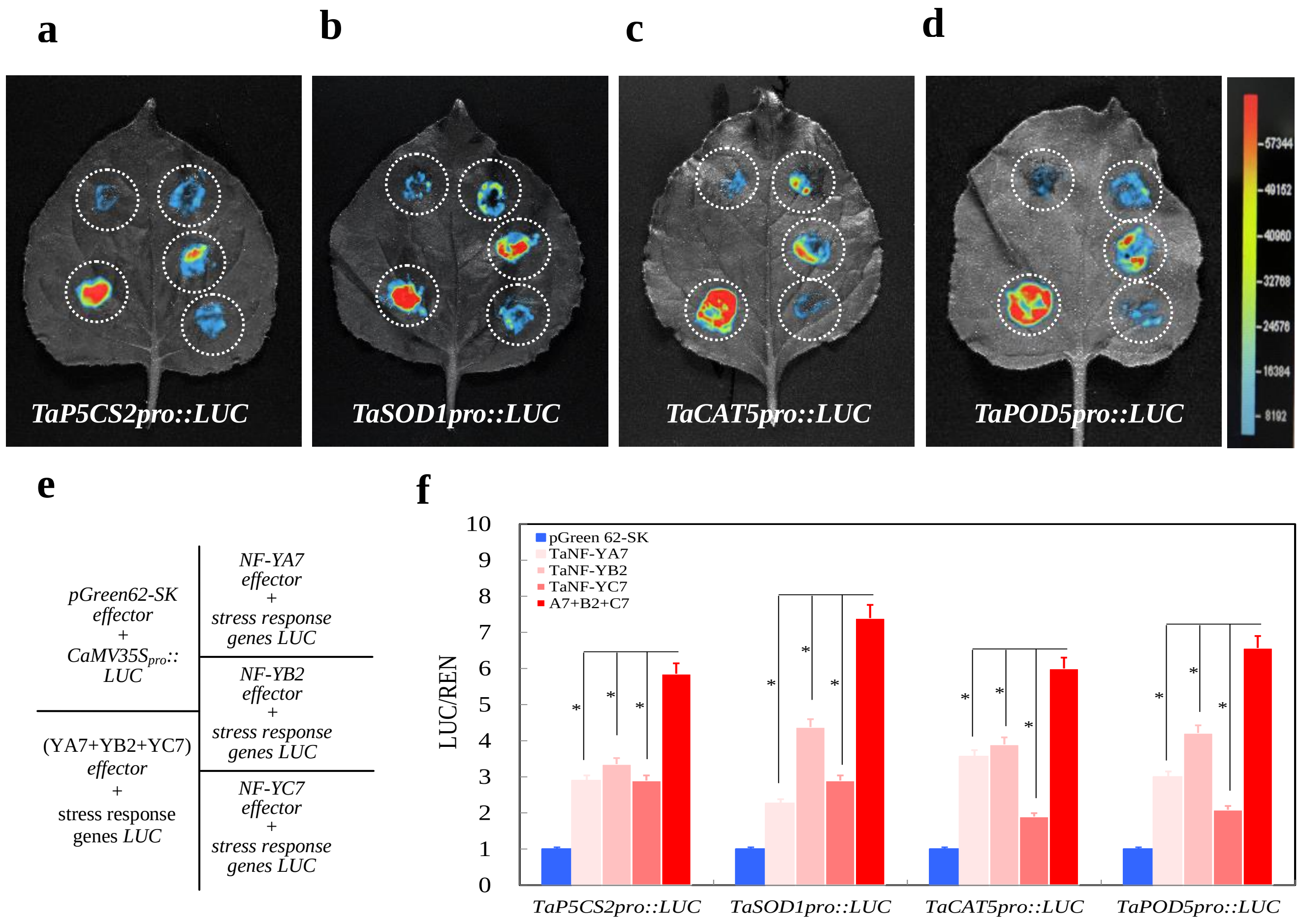

Fig. S10 Transcriptional activation assay results for distinct osmotic stress-associated genes regulated by TaNF-YB2, TaNF-YA7, and TaNF-YC7 Complexes.

(a)-(e) Dual-luciferase assays demonstrate that TaNF-YB2, TaNF-YA7, TaNF-YC7, and TaNF-YB2+TaNF-YA7+TaNF-YC7 transcriptionally activates *TaP5CS2*, *TaSOD1*, *TaCAT5*, and *TaPOD5*. (f) Cooperative and additive effects with TaNF-YB2, TaNF-YA7, TaNF-YC7 in driving *TaP5CS2*, *TaSOD1*, *TaCAT5*, *TaPOD5* expression. Luciferase activity is shown as LUC/REN ratio, using tobacco leaves co-transformed with the effector and the reporters. In (f) data presented are means ± standard deviation (n = 3). Student′s *t*-test was used to test the statistical significance (* p <0.05) between epidermal cells transformed with empty vector and cassette combinations.
